# Biodiversity and biocatalyst activity of culturable hydrocarbonoclastic fungi isolated from Marac–Moruga mud volcano in South Trinidad

**DOI:** 10.1038/s41598-021-98979-6

**Published:** 2021-09-30

**Authors:** Amanda C. Ramdass, Sephra N. Rampersad

**Affiliations:** Biochemistry Research Laboratory (Rm216), Department of Life Sciences, Faculty of Science and Technology, The University of the West Indies, St. Augustine, Trinidad and Tobago

**Keywords:** Biological techniques, Microbiology, Molecular biology, Environmental sciences

## Abstract

Mud volcanoes (MVs) are visible signs of oil and gas reserves present deep beneath land and sea. The Marac MV in Trinidad is the only MV associated with natural hydrocarbon seeps. Petrogenic polyaromatic hydrocarbons (PAHs) in its sediments must undergo biogeochemical cycles of detoxification as they can enter the water table and aquifers threatening ecosystems and biota. Recurrent hydrocarbon seep activity of MVs consolidates the growth of hydrocarbonoclastic fungal communities. Fungi possess advantageous metabolic and ecophysiological features for remediation but are underexplored compared to bacteria. Additionally, indigenous fungi are more efficient at PAH detoxification than commercial/foreign counterparts and remediation strategies remain site-specific. Few studies have focused on hydrocarbonoclastic fungal incidence and potential in MVs, an aspect that has not been explored in Trinidad. This study determined the unique biodiversity of culturable fungi from the Marac MV capable of metabolizing PAHs in vitro and investigated their extracellular peroxidase activity to utilize different substrates ergo their extracellular oxidoreductase activity (> 50% of the strains decolourized of methylene blue dye). *Dothideomycetes* and *Eurotiomycetes* (89% combined incidence) were predominantly isolated. ITS rDNA sequence cluster analysis confirmed strain identities. 18 indigenous hydrocarbonoclastic strains not previously reported in the literature and some of which were biosurfactant-producing, were identified. Intra-strain variability was apparent for PAH utilization, oil-tolerance and hydroxylase substrate specificity. Comparatively high levels of extracellular protein were detected for strains that demonstrated low substrate specificity. Halotolerant strains were also recovered which indicated marine-mixed substrata of the MV as a result of deep sea conduits. This work highlighted novel MV fungal strains as potential bioremediators and biocatalysts with a broad industrial applications.

## Introduction

Trinidad (10.6918° N, 61.2225° W) lies just 11 km northeast of Venezuela in the South American continent and is the last island in the Lesser Antilles volcanic island arc system of the West Indies^1^. Trinidad is the largest producer of oil and natural gas in the Caribbean and has a long history of exploration and production which began in 1857^2^. The main inland hydrocarbon reservoirs are located in the southern regions of the island. Controlled simulated assessments of various microbes to degrade petroleum hydrocarbons isolated from different natural seepages in Trinidad have been carried out^3–6^. Mohammed et al*.*^3^ concluded that specific microorganisms, which were indigenous to Trinidad, out-performed two commercial microbial-based bioremediation products used to reduce oil and grease content in a simulated, small-scale trial. Interestingly, Schulze-Makuch et al*.*^7^ reported that the unique microbial ecology (Bacteria and Archaea) of Trinidad’s Pitch Lake, located in La Brea in southwest Trinidad, the largest natural deposit of asphalt in the world, can be considered as a “terrestrial analogue” for understanding the biotic potential of hydrocarbon lakes located on Titan—Saturn's largest moon.

Polyaromatic hydrocarbons (PAHs) are highly recalcitrant compounds that create toxic environments that support growth and survival of adapted microorganisms usually at the species- or strain-specific level^8–12^. The United States Environmental Protection Agency has listed 16 PAHs as priority PAH pollutants^13^. With soil as a natural sink for petrogenic PAHs, high molecular weight (HMW) PAHs retained in soil particles and are of great interest especially since their migration into the multimedia environment can lead to human exposure. HMW PAHs do not undergo abiotic mechanisms or volatilization like the lower weight PAHs, and biological degradation influences their fate in the soil^14^.

Studies on fungal bioremediation of hydrocarbon-contaminated soils has been documented. Fungi possess several metabolic features that are advantageous to remediation but, their potential use in bioremediation has not been as explored as their bacterial counterparts. Additionally, few studies link PAH fungal consumption with chronically polluted natural hydrocarbon seeps spiked with PAHs such as MVs. Fungi are capable of transforming a wide range of organic pollutants^15–19^. The majority of fungal pollutant-degraders belong to *Ascomycota* and *Basidiomycota* phyla primarily, followed by subphylum *Mucoromycotina*^20^. Such fungi carry out intracellular hydroxylation of PAHs that produce water-soluble products which are then excreted^21–23^. Other studies have reported the use extracellular or secreted oxidoreductases by specific fungal strains that mineralize very carcinogenic, high-molecular-mass PAHs e.g. benzo[a]pyrene^22,23^. Harms et al*.*^20^ identified some of the unique characteristics of fungi with wide-ranging industrial applications viz. fungi-derived extracellular enzymes particularly those oxidoreductases that catalyse a broad range of substrates^24^. Studies on the use of native extracellular peroxidases as biocatalysts in detoxifying a number of chemicals including PAHs, have been described^25–27^. There are also reports of the development of recombinant peroxidases towards improving catalytic function^26–28^. Further, Marcano et al*.*^29^ explained the ability of *Fusarium alkanophilum* to extract water from light hydrocarbons which enables this fungal species to thrive in water-deficient hydrocarbon matrices; perhaps debunking the absolute requirement of water for life.

The terrestrial geothermal system referred to as "mud volcanoes" or “mud domes” are unique geological sites. The mud slurry exudate that is extruded from mud volcanoes (MVs) can be several kilometres deep and as such, the mud that erupts may be only slightly warmer than the ambient ground temperature (low temperature or cold seeps)^30^, but there can be steep temperature gradients and variable seepage rates^31^. Microbial community members were found to be compartmentalized into stratified niches along geochemical gradients of these MVs^32^. Unfortunately, such characterizations were made for bacteria and Archaea but, not for fungi. It is also important to note that identified microorganisms may have been introduced into the MV exudates from surface water (rain water) or due to a seasonal high groundwater table^7^. MVs are one of the visible signs of the presence of oil and gas reserves buried deep beneath land and sea and their distribution is strongly associated to the distribution of the world's petroleum assets as was reported for the South Caspian sedimentary basin^33^.

In Trinidad, MVs are mainly located in the forested regions of the Southern Range^34^ (The University of the West Indies, Seismic Research Institute http://uwiseismic.com/). Fewer MVs occur in the Central Range of the island and there are none in the northern parts of the country^34,35^. The MVs in Trinidad are not restricted to land as there are several that appear as “temporary islands” originating from the sea floor in Erin Bay^36^. To date, of the known eleven MVs, only the Marac MV located in Moruga, South Trinidad (10.0956° N, 61.3407° W) is associated with naturally-occurring hydrocarbon seeps. Battani et al*.*^37^ reported that the hydrocarbon fluids expelled from such MVs can be regarded as reflecting the present state of the hydrocarbons produced at depth, but they are not necessary directly representative of the hydrocarbons trapped at depth. More recent geological surveys, conducted after the August 21st 2018 earthquake fault rupture, revealed seven new MVs with evidence of hydrocarbon gas expulsion in Los Iros along the island’s southwestern coast. These MVs in Trinidad are also paleontologically significant^38^.

This study determined the biodiversity of culturable fungi from soil (i.e. mud and oil) sampled in an unexplored MV in South Trinidad and investigated the extracellular peroxidase activity of such isolated fungal species to utilize different substrates with the potential for commercial production as biocatalysts. Additionally, the study highlighted the occurrence of putative PAH-degrading fungi and established PAH metabolic potential in vitro of the culturable fungi in the MV and the occurrence of halotolerant fungi, some with the ability to produce biosurfactant compounds.

## Results

### Isolate recovery

A total of 67 fungal colonies were isolated from the Marac MVs. Of these, 47 were deemed as morphologically different and were included in the study (Table 1).

**Table 1:** MV sampling and fungal isolation data from the four geographically separated sites. *One isolate is a yeast-bacterium co-culture.

Colonies isolated	No. of fungi recovered		Total
	Plating study	Enrichment	
Overall	34	33	67
**Unique colonies selected per sample type**			
M1 (Inside the volcano’s vent)	3	3	6
M2 (Edge of the volcano’s vent)	5	5	10
M3 (Edge of the volcano’s vent)	10	7	17
M4 (Edge of the vegetation margin with oil)	8	6	14
Total unique isolates	26	21	47
Oil-degrading isolates			37*
Top isolates			19*

### Growth and oil-tolerance of axenic fungi

**Figure 1:**
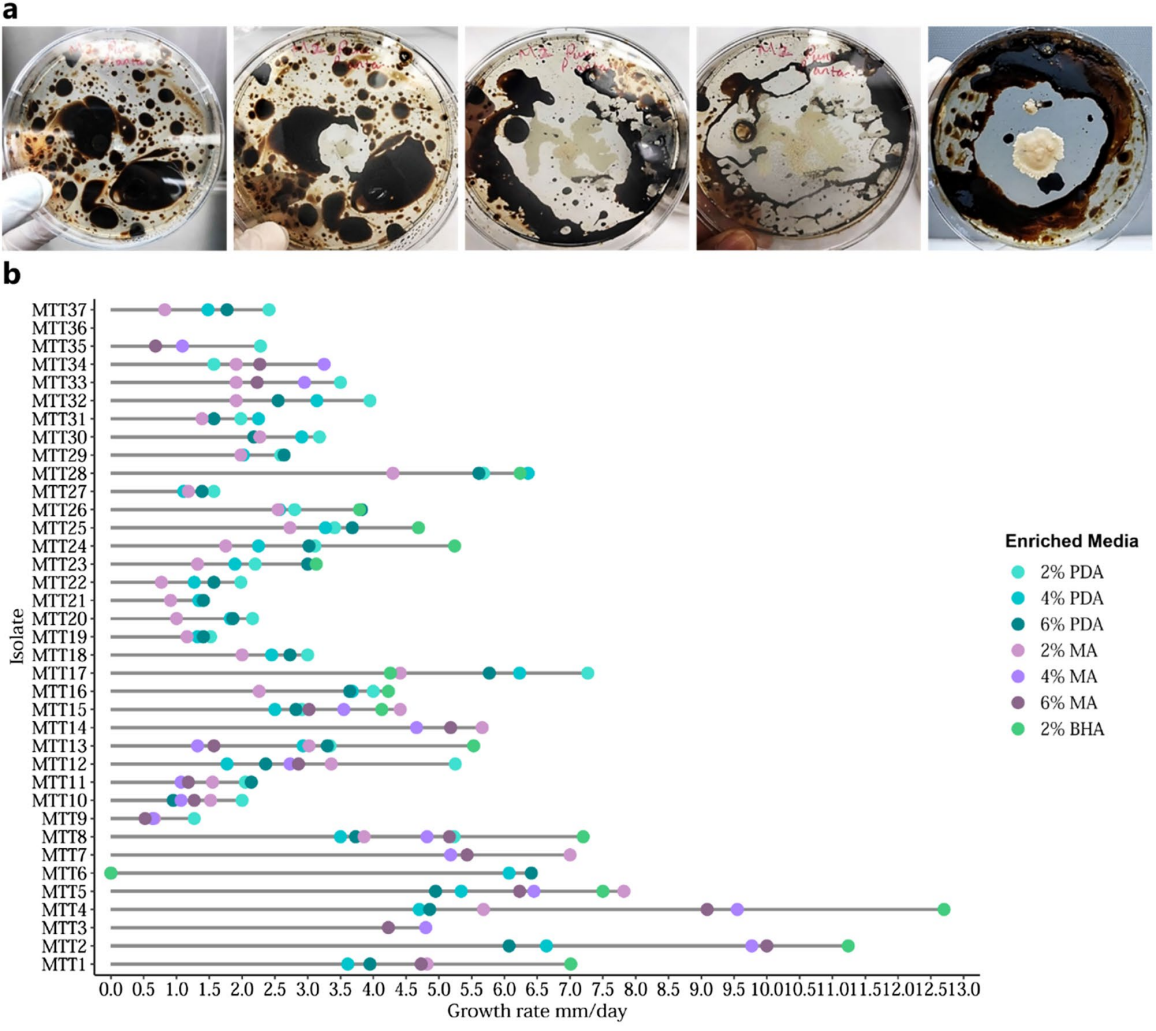
Growth on oil-amended media. (**a**) Performance of yeast-bacterium co-culture on crude oil enriched media; left to right 2% oil plates after 0, 24, 48, 72 h and 4% oil plate after 72 h. (**b**) Growth rate on respective crude oil enriched media (RStudio software v1.3.1093).

From the 47 isolates, 37 isolates, inclusive of a co-isolated yeast-bacterium that showed exceptional performance on oil enriched media (Fig. 1), were capable of metabolising crude oil based on their growth/performance on crude oil enriched media (2%) (Table 1). The isolates were divided into two groups: group A, top-performers, which included 18 isolates with the highest oil tolerance (≥ 3 mm/day growth rate on 6% crude oil) and group B which included 18 isolates with lower tolerance (see Supplementary Table S1). To emphasize the utility of crude oil as a sole carbon source, group A isolates were screened on BHA supplemented with crude oil (2%) as the sole carbon source. All were able to grow and utilized crude oil as the sole carbon source (see Supplementary Table S1).

Growth rates were determined for the 37 isolates at various concentrations of crude oil on different media (Fig. 1, see Supplementary Table S1). There was no significant difference (α = 0.05; *P* = 0.429) in fungal growth on PDA and MA media amended with 2%, 4% and 6% crude oil. The isolates with the highest tolerance potential on PDA were observed for *Montagnula opulenta* MTT17, *Fusarium equiseti* MTT2 and *Aspergillus flavus* MTT6 at 2%, 4% and 6% crude oil, with growth rates of 7.27, 6.64 and 6.41 mm/day, respectively, and on MA for *Aspergillus flavus* MTT5, *Fusarium equiseti* MTT2 and *Fusarium equiseti* MTT2 at 2%, 4% and 6% crude oil, with values 7.82, 9.77, 10.00 mm/day, respectively. The isolates with the lowest tolerance potential on PDA were observed for *Cladosporium dominicanum* MTT9, *Cladosporium cladosporioides* MTT27 and *Cladosporium dominicanum* MTT9 at 2%, 4% and 6% crude oil, with growth rates of 1.27, 1.11 and 0.64 mm/day, respectively and on MA for *Aspergillus flavus* MTT6, at 2%, 4% and 6% crude oil, with no growth observed. Overall, the isolates showed the highest radial growth on 2% crude oil (75.68% isolates) followed by 6% crude oil (16.22% isolates) then 4% crude oil (8.11% isolates). Taken together all these data confirm the utilization of crude oil by all 37 isolates. There was no significant difference in growth on PDA amended with 2%, 4% and 6% crude oil and for MA with 2% crude oil according to Fisher’s Pairwise comparisons. This data can be viewed in Supplementary Table S2.

Several isolates belonging to the same species demonstrated a different growth response in the presence of crude oil, which may indicate intraspecific variability. For example, regarding the species *Aspergillus flavus*, strain MTT5 was able to grow at all concentrations of oil (2%, 4%, 6%) while the MTT6 strain was inhibited at 2% MA, 2% BHA, 4% MA and 6% MA. There were also differences in the growth response of the same species on the same media and concentration of oil as evidence of intraspecific variability. For example, *Cladosporium cladosporioides* MTT10, MTT22, MTT27, MTT35 strains exhibited maximum growth on the lowest concentration, whereas MTT11, MTT21, MTT23 exhibited maximum growth on the highest concentration; *Penicillium oxalicum* MTT32 showed the highest growth rate on 2% PDA crude oil whereas MTT31 showed highest growth on 4% PDA. Also, MTT32 had higher growth rates for all concentrations of oil than MTT31; *Penicillium citrinum* MTT25, MTT26 and MTT29 displayed the highest growth rates on the highest concentration of crude oil versus MTT30 which displayed the lowest growth rate on the highest concentration of crude oil and the highest on the lowest concentration. Also, higher growth rates were observed for a given media, either PDA or MA. For example, *Neoscytalidium hyalinum* MTT3, MTT7, MTT14 all had equal performance on 2%, 4% and 6% PDA where the colony was overgrown within the allotted incubation period but growth on MA was reduced substantially. Despite variations in the growth rates on the various concentrations of crude oil-amended media, most isolates were capable of growth suggesting that there was no potential inhibitory effect at higher levels of crude oil.

The enrichment procedure revealed the presence of secreted compounds with potential biosurfactant activity. For several isolates, after 4 days of incubation at 37 °C at 200 rpm, perfectly spherical oil globules were observed (see Supplementary Fig. S1). Negative controls consisted of (i) crude oil only in PDB, and (ii) Tween 20 with crude oil only in PDB. Tween 20 is a polyoxyethylene sorbitol ester and functioned as a non-ionic detergent to disperse the crude oil on the surface of the PDB. During the initial incubation period, approximately 20–40% of the crude oil's mass is lost due to conversion to gases and dissolution of water-soluble hydrocarbons into the PDB. The remaining fraction is composed of more viscous compounds that retard the spread of oil on the surface of the PDB resulting in the formation of visible oil clumps. Agitation of the PDB cultures during the incubation period may also contribute to oil clumping^39^. However, if the physical/mechanical manipulations of the broth were a factor in this study, then all broth cultures would have developed these oil globules. Thus, they were attributed to the secreted dispersants produced by the fungal isolates in these broth cultures.

### Identification of hydrocarbonoclastic fungi

**Figure 2:**
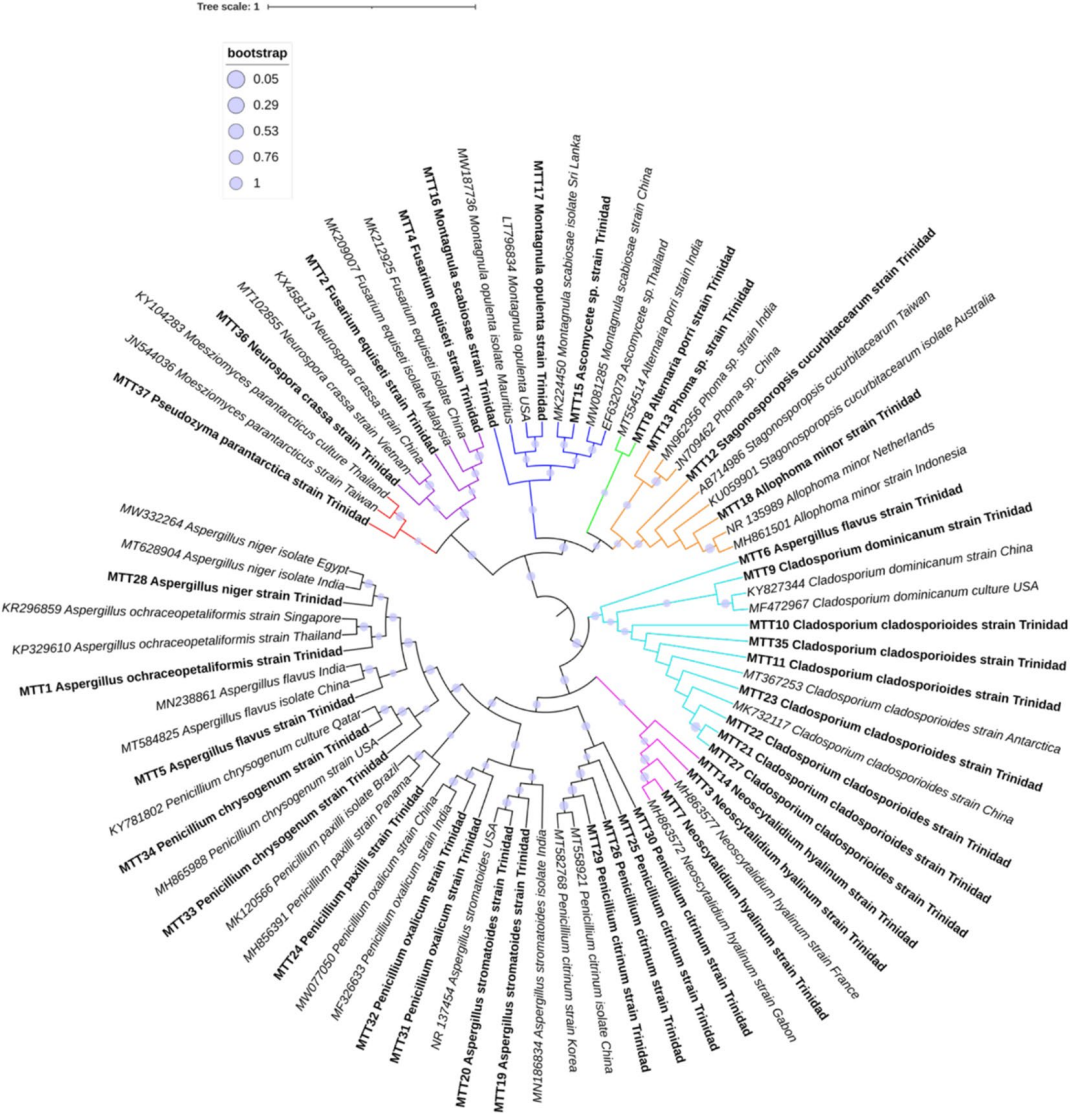
Un-rooted Maximum Likelihood phylogenetic tree for filamentous fungi. Among the 37 fungal species detected in this study, only three fungi (*Aspergillus*, *Fusarium* and *Penicillium*) at the genus level have been previously reported as being isolated from crude oil-contaminated sites in Trinidad^6^. The findings of this study described the first report of six novel oil-degrading fungi including *Aspergillus ochraceopetaliformis*, *Fusarium equiseti*, *Neoscytalidium hyalinum/dimidiatum*, *Alternaria porri*/*destruens*, *Montagnula scabiosae* and *Montagnula opulenta*. Additionally, previously identified species from other studies of *Aspergillus flavus*, *Phoma* sp., *Cladosporium cladosporioides*, *Penicillium citrinum*, *Penicillium paxilli* and *Aspergillus niger* were identified for the first time in Trinidad. (iTOL v6; https://itol.embl.de/).

Sequence comparisons revealed the identities of those isolates capable of growth in vitro on crude oil-amended media (see Supplementary Table S3). Identification was based on ITS and 16S sequence (to determine the bacterium in the yeast-bacterium co-culture) comparisons with consensus sequences in the GenBank database. Where applicable, additional markers supported the ITS sequence identities. Un-rooted phylogenetic trees using the Maximum Likelihood method based on the General Time Reversible (GTR + G + I) as the best fit model with 1000 bootstrapped replicates was used to perform sequence analysis. Phylogenetic analysis indicated moderate (> 75%) to high (> 90%) bootstrap support. The identities of the Trinidad isolates were, therefore, confirmed at the genus level for all sequences and at the species level for the majority of the sequences (Fig. 2, see Supplementary Table S3).

### Diversity and taxonomic composition

Abundances of fungal taxa were examined at a class, genus and species level to determine the α-diversity and composition of the hydrocarbonoclastic fungal communities. α-diversity indices can be found in Table 2. Shannon’s diversity indices (H) observed indicated a high fungal diversity and the Simpson’s dominance index (D_2_) reflected uneven communities among genera and species present where most of the taxa were equally present, and a community that was split for the classes detected. The abundance corresponded to the Zipf Model (see Supplementary Fig. S2) and the Akaike information criterion (AIC) data can be viewed in Supplementary Table S4.

**Table 2:** Diversity indices for the hydrocarbonoclastic fungal community.

Taxonomic level	Diversity		Richness		Dominance	
	H	D_1_	*S*	Chao 1	Absolute	D_2_
Class	1.020	0.592	4	4.000	18	0.408
Genus	2.170	0.850	13	25.200	9	0.150
Species	2.820	0.922	21	37.900	7	0.078

**Figure 3:**
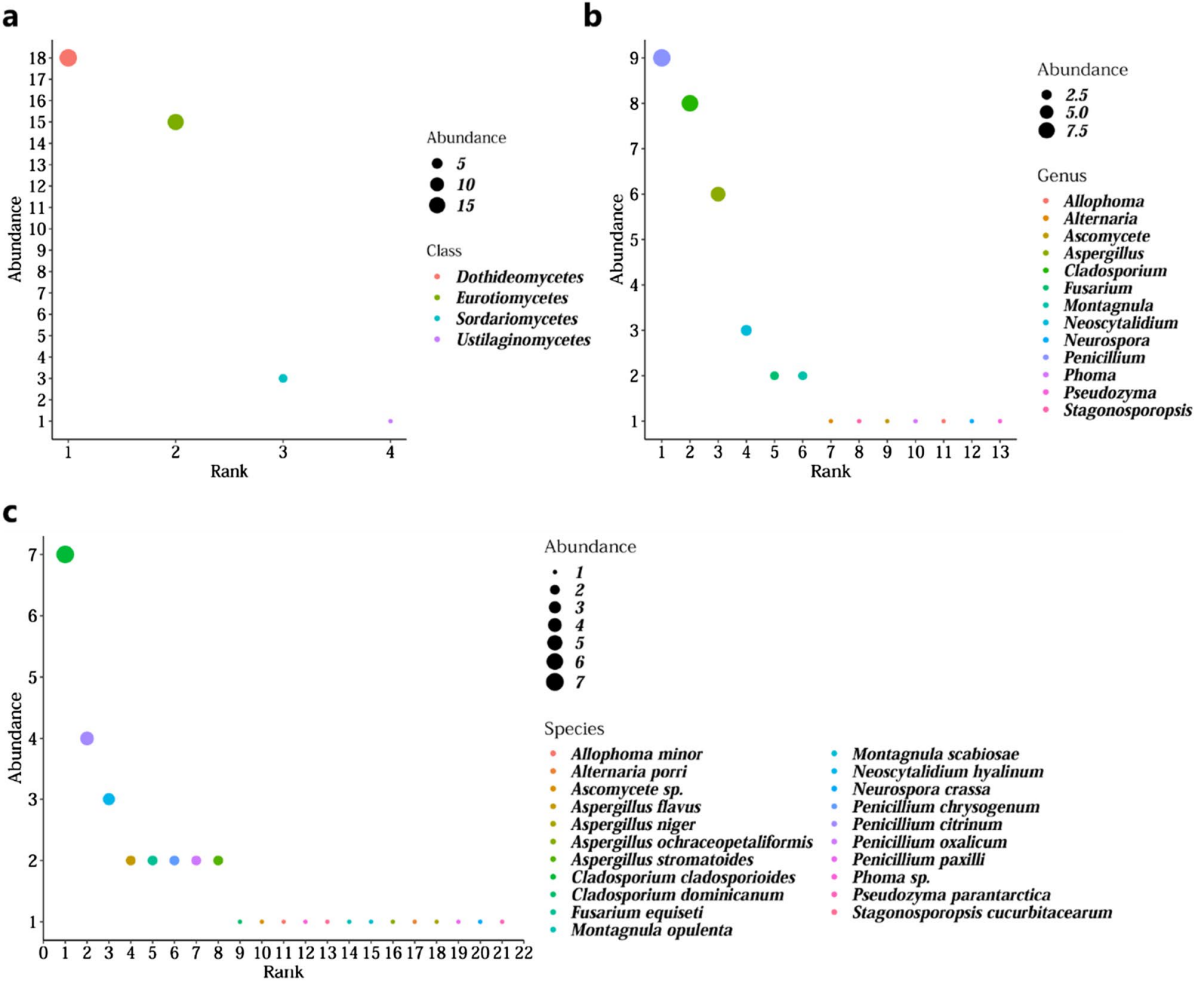
Ranked abundance for fungal (**a**) classes, (**b**) genera and (**c**) species. (RStudio software v1.3.1093).

Taxa of specific classes, genera and species dominated the fungal community (Fig. 3, see Supplementary Table S5). The predominant class of fungi were *Dothideomycetes* and *Eurotiomycetes* occupying 89% of the total community. *Sordariomycetes* and *Ustilaginomycetes* were detected at low abundances, 8.11% and 2.70% respectively. The genera *Penicillium*, *Cladosporium* and *Aspergillus* were detected with the highest abundances accounting for 24.20%, 21.62% and 16.22%, respectively, of the community composing of the species *Penicillium citrinum*, *Penicillium chrysogenum*, *Penicillium oxalicum*, *Penicillium paxilli*, *Cladosporium cladosporioides*, *Cladosporium dominicanum*, *Aspergillus flavus*, *Aspergillus stromatoides*, *Aspergillus ochraceopetaliformis* and *Aspergillus niger*. Less abundant genera including *Neoscytalidium* (8.11%) consisting of the species *Neoscytalidium hyalinum* followed by *Fusarium* and *Montagnula* (5.41% each) consisting of the species *Fusarium equiseti*, *Montagnula opulenta* and *Montagnula scabiosae* were isolated. Even less abundant genera *Alternaria*, *Stagonosporopsis*, *Ascomycete*, *Phoma*, *Allophoma*, *Neurospora* and *Pseudozyma* each accounting for 2.70% of the fungal community, were detected with only one individual each. The species detected included *Alternaria porri*, *Stagonosporopsis cucurbitacearum*, *Ascomycete* sp*.*, *Phoma* sp*.*, *Allophoma minor*, *Neurospora crassa* and *Pseudozyma parantarctica*.

### Biosurfactant detection

The top-performing 18 isolates in group A, were screened for biosurfactant production. The broth cultures of 12 isolates showed positive results in the oil-spreading assay and could spread the paraffin oil immediately upon contact with the culture media, thereby indicating the presence of compounds with biosurfactant activity. The remaining fungal cultures did not result in positive oil-dispersion despite the addition of more broth to increase the biosurfactant load. Further screening and characterization should be performed to identify and characterize the biosurfactant(s) produced. Table 3 outlined the biosurfactant activity of the fungal strains.

**Table 3:** Biosurfactant activity of the selected isolates. *Biosurfactant detection: (+) biosurfactant produced; (−) no biosurfactant produced.

Isolate	Species	Biosurfactant*
MTT1	*Aspergillus ochraceopetaliformis/flocculosus*	+
MTT2	*Fusarium equiseti/incarnatum*	+
MTT3	*Neoscytalidium hyalinum/dimidiatum*	+
MTT4	*Fusarium equiseti/incarnatum*	+
MTT5	*Aspergillus flavus*	+
MTT6	*Aspergillus flavus*	+
MTT7	*Neoscytalidium hyalinum/dimidiatum*	−
MTT8	*Alternaria porri/tenuissima/destruens*	+
MTT13	*Phoma* sp.	−
MTT14	*Neoscytalidium hyalinum/dimidiatum*	+
MTT15	*Ascomycete* sp.	+
MTT16	*Montagnula scabiosae*	+
MTT17	*Montagnula opulenta*	+
MTT23	*Cladosporium cladosporioides/tenuissimum/colombiae/oxysporum*	−
MTT24	*Penicillium paxilli*	+
MTT25	*Penicillium citrinum*	−
MTT26	*Penicillium citrinum*	−
MTT28	*Aspergillus niger*	−

### Secreted oxidoreductase activity

**Figure 4:**
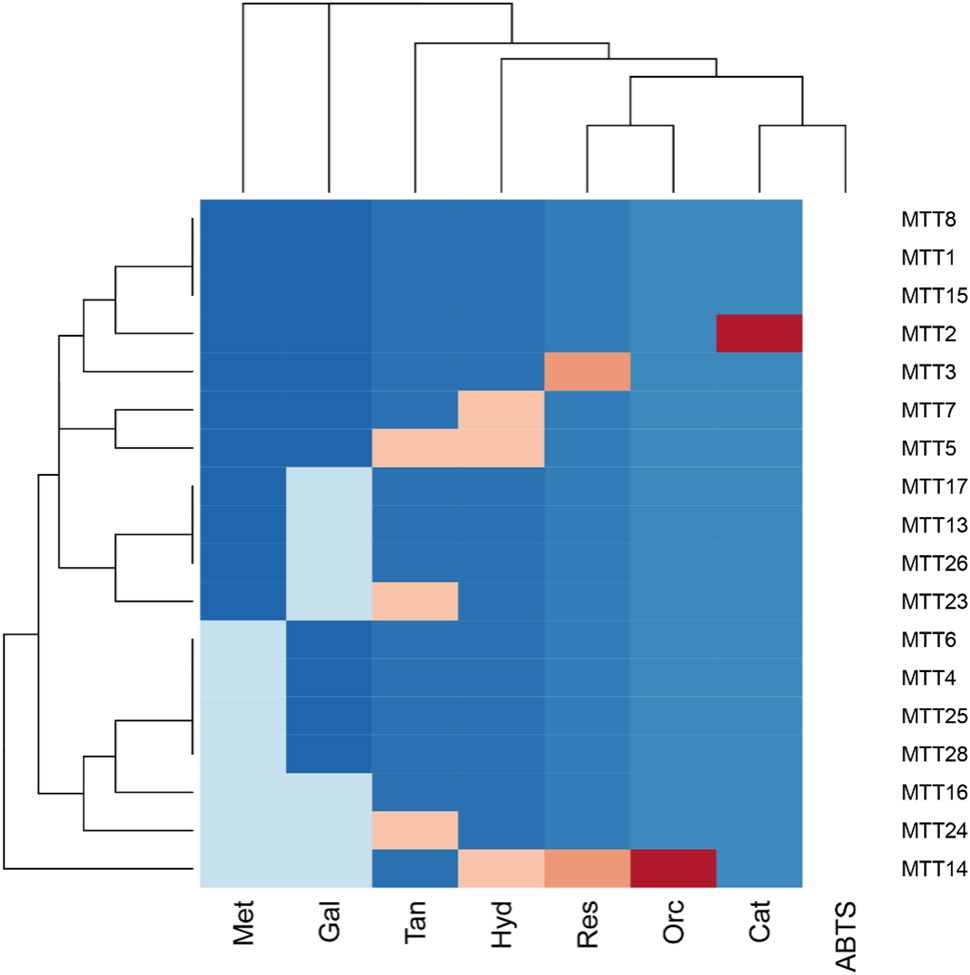
Substrate spectrum of secreted fungal oxidoreductases. *Met* methylene blue, *Gal* gallic acid, *Tan* tannic acid, *Hyd* hydroquinone, *Res* resorcinol, *Orc* orcinol, *Cat* catechol, 2,2′-azino-bis-(3ethylbenzothiazoline-6-sulfononic acid) diammonium salt = ABTS. ABTS was the only substrate where oxidation was detected in all the strains (white column). Darker blue blocks indicate groupings where for a given substrate, there was oxidation for the same number of isolates (for example, the darkest blue columns show that for substrates Meth and Gal, 11 strains showed activity). Groupings for a given substrate where no oxidation for the same number of isolates is shown in light blue, orange and red blocks (for example, a red block shows one fungal strain having no activity; light blue blocks show 7 strains with no activity). The dendrograms provide an indication to the relationship between isolates and oxidation of the substrates (RStudio software v1.3.1093).

Fungal strains were tested to determine the substrate spectrum of secreted fungal oxidoreductases in crude enzyme extracts^40^. Oxidoreductase activity among 18 top-performers was screened using 8 substrates grouped into 5 categories which were: aromatic carboxylic acids, aromatic alcohols, aromatic azo dyes, polyphenols and thiazine dye (Table 4, Fig. 4). Those substrates that underwent oxidation by secreted oxidoreductases that resulted in a change in UV spectrum were marked recorded as positive for activity. Control reactions lacking an enzyme source were performed in parallel which excluded the prospect of non-enzymatic oxidation. *Aspergillus ochraceopetaliformis* MTT1, *Alternaria porri*/*tenuissima/destruens* MTT8 and *Ascomycete* sp. MTT15 were the most versatile strains as reflected by the broadest substrate spectrum where oxidation was detected for all 8 substrates. *Neoscytalidium hyalinum/dimidiatum* MTT14 had the most limited substrate range with only 3 oxidized substrates; however, the two other *Neoscytalidium* strains MTT3 and MTT7 oxidized 7 substrates. 2,2′-azino-bis-(3ethylbenzothiazoline-6-sulfononic acid) diammonium salt (ABTS) is one of the most commonly used substrates to screen for oxidases^41^ and was the only substrate where oxidation was detected in all the strains (see Supplementary Table S6). The isolate with the highest activity for a given substrate is highlighted in Table 4. Overall, the substrates with the most to least activity detected across all the fungal strains were ABTS > catechol = orcinol > resorcinol > hydroquinone = tannic acid > gallic acid = methylene blue (Fig. 4).

**Table 4:** Fungal isolates with the highest extracellular oxidoreductase activity for each of the 8 tested substrates (ChemBioDraw v12.0, level: ultra).

No.	Category	Compound		Highest activity
1	Aromatic carboxylic acids	Gallic acid		*Fusarium equiseti* MTT4
2	Aromatic alcohols	Catechol		*Montagnula opulenta* MTT17
3		Hydroquinone		*Alternaria porri* MTT8
4		Resorcinol		*Aspergillus flavus* MTT6
5		Orcinol		*Ascomycete sp.* MTT15
6	Aromatic azo compound	ABTS		*Montagnula scabiosae* MTT16
7	Polyphenols	Tannic acid		*Alternaria porri* MTT8
8	Thiazine dye	Methylene blue		*Aspergillus ochraceopetaliformis* MTT1

## Discussion

Microbial decomposition of petroleum hydrocarbon compounds has been demonstrated to be one of the more operative bioremediation approaches available^42^. However, little is known about the crude oil degradation potential of fungi in Trinidad, which have had a longstanding existence in chronically polluted terrestrial habitats. Additionally, the Marac MV is a unique geological site that can give an indication of the deep subsurface biosphere which would be selectively enriched for fungal species (i) with extremely high tolerance to hydrocarbon pollution, (ii) capable of growth in an extreme or hostile environment that interferes with oxygen, water and nutrient availability as well as withstanding the toxic effects of oil-constituents, especially aromatic compounds and (iii) that formulate exclusive cooperative relationships with other microbes^20,43,44^. This research identified several novel fungal strains as potential candidates for bioremediation strategies and biotechnological applications.

In this study, 37 fungal isolates were proven to be hydrocarbonoclastic. Isolates in group A had a high oil-tolerance and isolates in group B had lower oil-tolerance but showed carbon intake adequate for lower-level growth. Marac MVs discharged saline water that migrated through hydraulic fracture systems. As such, the fungal strains isolated in this study may also be considered to be halotolerant strains as they demonstrated growth on marine media. It was reported that halotolerant strains were isolated from oil-contaminated tropical soil^45^ and sodium chloride-amended hydrocarbon media^46^. Halotolerant bacterial strains have been identified in other MVs in Trinidad with specific biogeochemical capabilities including decomposition of linear aliphatic and aromatic compounds^7^.

The measure of microbial communities and enzymes in soil have been established as indictors of biological activity and pollution^47,48^ where abundances of fungal taxa can indicate the changes occurring within contaminated soil^47,49^. The composition of recovered fungi was expectedly dominated by *Ascomycota*, the most abundant phylum recovered from chronically polluted hydrocarbon-contaminated soil^50^, followed by *Basidiomycota*. While pollutant degraders are known to belong to these phyla, detection of others are less commonly reported^20^. In total, 4 fugal classes were identified including *Dothideomycetes* and *Eurotiomycetes* which were the largest taxonomic classes detected (48.65% and 40.54% respectively), and *Sordariomycetes* and *Ustilaginomycetes* in considerably lower abundances (8.11% and 2.70% respectively). At a deeper taxonomic level, 13 genera were detected including *Penicillium*, *Cladosporium*, *Aspergillus*, *Neoscytalidium*, *Fusarium*, *Montagnula*, *Alternaria*, *Stagonosporopsis*, *Ascomycete*, *Phoma*, *Allophoma*, *Neurospora* and *Pseudozyma*. Among the genera, the fungal candidates with the highest oil-tolerance in group A were affiliated to the species *Aspergillus ochraceopetaliformis*, *Fusarium equiseti/incarnatum*, *Neoscytalidium hyalinum/dimidiatum*, *Aspergillus flavus*, *Alternaria porri*/*tenuissima/destruens*, *Phoma* sp., *Ascomycete* sp., *Montagnula scabiosae*, *Montagnula opulenta*, *Cladosporium cladosporioides*, *Penicillium paxilli, Penicillium citrinum* and *Aspergillus niger*. Abundance and proportions of each taxonomic group detected can be viewed in Supplementary Table S5.

Despite the high biodiversity of the hydrocarbonoclastic fungal community (genera: H = 2.17, D_1_ = 0.85; species: H = 2.82, D_1_ = 0.92), specific genera were found to dominate the community including *Penicillium* (24.32%), *Cladosporium* (21.62%) and *Aspergillus* (16.22%), all of which are well-known degraders of hydrocarbons^20,49,51–54^. Importantly, Rank-abundance dominance (RAD) analysis showed that the distribution of abundances within the fungal community fit the Zipf model which indicated the dependence of species on physical conditions and the presence of other species^55^. The crude oil bioassays revealed a unique yeast-bacteria co-culture showing exceptional oil-degrading potential. The survival cost for pioneer species is low but high for late successional species (viz. energy, time and adaptation before they can thrive) and thus these species can be considered as rare^42,55^.

Hydrocarbonoclastic fungi isolated in this study, were linked to known PAH and/or hydrocarbon degraders. *Aspergillus flavus* can degrade kerosene^56^, aviation fuel^57^, benzo[a]pyrene, a 5-ring priority PAH and one of the most important industrial pollutants^58^ and six other PAHs among the 16 priority PAHs^59^. *Phoma* sp. isolated from petroleum-contaminated soil could degrade hydrocarbons^60^ including phenanthrene^61^. *Cladosporium cladosporioides* has been isolated from chronically polluted soil ^58^ and studied for its extracellular oxidising activity^62,63^. *Penicillium citrinum*, frequently isolated from oil-contaminated soil^64^, can efficiently reduce crude oil content by 77% and individual *n*-alkane fractions by an average of 95.37%, albeit as marine substrates^65^ and has the ability to degrade aviation fuel^57^. *Aspergillus niger*, being a hydrocarbon-utilizer^53,60^, can degrade the aromatic compounds styrene and toluene^17^, aviation fuel^57^, remediate crude oil-polluted soil effectively, is a good biostimulant^66^ and has been shown to produce lipolytic enzymes^67^ and laccases^68^. *Penicillium paxilli*, isolated from soil^69^, was reported as a fungus with value-added products in applications such as food, pharmaceuticals, cosmetics and paper industries^70^, but it has not been widely reported for hydrocarbon degradation except for aviation fuel^57^ and aquatic pelagic tars^71^. Therefore, the isolation of these fungal strains in our study adds a valuable contribution to the field of indigenous fungi in bioremediation. In Trinidad, these species are novel, being reported for the first time.

*Aspergillus ochraceopetaliformis*, *Fusarium equiseti*, *Neoscytalidium hyalinum/dimidiatum*, *Alternaria porri*/*destruens*, *Montagnula scabiosae*, *Montagnula opulenta* species have been reported here for the first time globally and in Trinidad as hydrocarbonoclastic fungi. *Aspergillus ochraceopetaliformis* is not commonly detected but has been isolated from soil in India, Venezuela, Slovenia, Greece and Costa Rica^72^ and studied for its antiviral activity^73^ and bioactive metabolites as potential anti-infective agents^74^; however, there are no published reports on its oil degradation potential and its use in bioremediation. While several species of *Fusarium* have been reported as oil-degraders^75^, *Fusarium equiseti* is reported here for the first time. *Fusarium incarnatum* has been isolated in a diversity study of oil contaminated soil^76^ and has been reported to produce lipase^77^ and laccase^78^.

*Neoscytalidium hyalinum/dimidiatum* and *Alternaria porri*/*destruens* are completely novel. *Alternaria tenuissima* has been isolated from oil contaminated water but was strongly inhibited by crude oil presence^79^ and has also been reported to degrade polyurethanes, an important compound in plastic degradation and recycling^80,81^. *Alternaria destruens* is a beneficial soil microbe that can promote the growth of plants^82^; this fungus then, can be further investigated for its benefits of vegetative growth in heavily contaminated soil as in Marac. Apart from the few studies that include *Montagnula scabiosae* in taxonomic and phylogenetic studies^83–85^, this species together with *Montagnula opulenta* are not reported in the literature for any impactful research use until now.

Among the 18 fungal isolates screened for biosurfactant production, 12 isolates: *Aspergillus ochraceopetaliformis* MTT1, *Fusarium equiseti* MTT2 and MTT4, *Neoscytalidium hyalinum/dimidiatum* MTT3 and MTT14, *Aspergillus flavus* MTT5 and MTT6, *Alternaria porri*/*tenuissima/destruens* MTT8, *Ascomycete* sp. MTT15, *Montagnula scabiosae* MTT16, *Montagnula opulenta* MTT17 and *Penicillium paxillin* MTT24, showed positive results, thereby indicating the presence of compounds with biosurfactant activity in the broth cultures. Morikawa et al.^86^ concluded that oil displacement is directly proportional to the concentration of biosurfactant(s) in solution. Microbial biosurfactant production serves two functions: (i) in emulsification (increases bioavailability of substrate) and (ii) reduction of cell surface hydrophobicity (enabling association of hydrophobic substrates), thereby enhancing degradation of petroleum hydrocarbons^20,87^.

Various microbes produce a range of biosurfactant compounds^88^, but those produced by fungi are less commonly reported in the literature compared to bacteria (see Supplementary Table S7 and the references therein). The functional impact of biosurfactant production also lies in surfactant-induced shifts in microbial community dynamics in chronically-polluted soil^89^. In this study, the following biosurfactant-producing strains were reported for the first time: *Aspergillus ochraceopetaliformis*, *Fusarium equiseti/incarnatum*, *Neoscytalidium hyalinum/dimidiatum*, *Alternaria porri*/*tenuissima/destruens*, *Ascomycete* sp., *Montagnula scabiosae*, *Montagnula opulenta* and *Penicillium paxillin*. The value of these novel indigenous strains lies in greater biodegradability, selectivity and low-ecotoxicity in comparison to their commercial counterparts^3,90,91^.

Hydrocarbonoclastic strains produce mono- and di- oxygenases that aid in initial oil degradation steps^92,93^ and support fungal growth on recalcitrant substrates^16^. As the second largest class of enzymes used in biotechnology, oxidoreductases have been linked to acceleration of degradation of hydrocarbons and there is a correlation between enzymatic redox activities in soil as an early indicator of oil degradation^94^. In this study, hydroxylase activity was recorded for fungal strains when ABTS as a non-phenolic substrate and gallic acid, catechol, hydroquinone, resorcinol, orcinol, tannic acid and methylene blue phenolic substrates were used. ABTS is one of the most commonly used substrates to assess phenol oxidase (EC 1.10.3.1) enzymes which catalyse the oxidation of xenobiotic and recalcitrant aromatic compounds^95–99^. Catechol and guaiacol are redox mediators that improve the efficiency catalysis of oxidation reactions by aiding in the transfer of electrons from the substrate^41,100^. Several fungi have been reported to demonstrate hydroxylase activity including: *Aspergillus* and *Penicillium* from soil—high ABTS catabolic activity^97,101,102^, *Cladosporium cladosporioides*—extracellular ABTS-oxidising activity^62,63^, *Trametes versicolor*, *Agaricus bisporus*, *Myceliophthora thermophila* and *Botrytis cinerea*—oxidized gallic acid and catechol^103,104^, *Aspergillus niger*—degradation of orcinol^105^, *Trametes versicolor*, *Agaricus bisporus* and *Myceliophthora thermophila*—transformation of tannic acid^103^.

Steric hindrance can contribute to the inefficient binding of the oxidoreductase enzyme to larger substrates like tannic acid^106^. Generally, the enantio-preference substitution pattern of phenols affects oxidoreductase activity^107^. The stereochemical effect of the hydroxyl group at the *meta*-position may explain difficulty in hydrolysis in compounds with this substitution pattern. The compounds that served as substrates for secreted oxidoreductase enzymes of the fungal isolates from Marac MV were those with OH-directors in either substitution pattern on the benzene aromatic ring structure^41^. Although there is a lack of understanding concerning how the benzene ring changes or interacts during the catalytic process^108^, the minimal structural requirements for enzyme effectors appear to be a 1,3-dihydroxybenzene^109^.

Hydroquinone is a benzene metabolite, with known haematotoxic and carcinogenic properties. Fungi capable of transforming or mineralizing hydroquinone include *Aspergillus fumigatus*^110^, *Candida parapsilosis*^111^, *Tyromyces palustris*, *Gloeophyllum trabeum*^112^, *Penicillium chrysogenum*^46^, *Phanerochaete chrysosporium*^113^, *Trametes versicolor*, *Agaricus bisporus* and *Myceliophthora thermophila*^103^. *Ortho*-substituted compounds (e.g. catechol and gallic acid) are better substrates than *para*-substituted compounds (e.g. hydroquinone), while the lowest rates are obtained with *meta*-substituted compounds (e.g. orcinol and resorcinol) and the highest activity is seen with the non-phenolic substrate ABTS^114^.

Industrial effluents contain a relatively high concentration of xenobiotic compounds, such as dyes, which are intractable to degradation and are toxic to aquatic species and humans^115,116^. Oxidoreductases are known decolourizers and are used in the degradation of a wide range of dyes. The activity of the enzyme is inhibited by excess hydrogen peroxide and this inhibition is blocked by tryptophan, an aromatic amino acid^117^. Various species of filamentous fungi were reported to decolorize methylene blue dye including *Rhizopus stolonifer*, *Aspergillus fumigates*, *Aspergillus niger*, *Fusarium solani* and *Penicillium funiculosum*^118^, *Coriolus versicolor*^119^, *Paraconiothyrium variabile*, *Trametes versicolor* and *Aspergillus oryzae*^120^ and the dimorphic yeast species *Galactomyces geotrichum* strain KL20A^116^.

In this study, oxidoreductase activity in the presence of tryptophan was recorded for all fungal strains where some showed activity for more substrates than others. *Aspergillus ochraceopetaliformis/flocculosus* MTT1, *Alternaria porri/tenuissima/destruens* MTT8 and *Ascomycete* sp. MTT15 had the broadest substrate spectrum where oxidation was detected for all 8 substrates tested and *Neoscytalidium hyalinum/dimidiatum* MTT14 had the most limited substrate range with only 3 oxidized substrates. Complete decolorization of methylene blue occurred at the end of 20 days by *Aspergillus ochraceopetaliformis/flocculosus* MTT1, *Fusarium equiseti/incarnatum* MTT2, *Neoscytalidium hyalinum/dimidiatum* MTT3 and MTT7, *Aspergillus flavus* MTT5, *Alternaria porri/tenuissima/destruens* MTT8, *Phoma* sp. MTT13, *Ascomycete* sp. MTT15, *Montagnula opulenta* MTT17, *Cladosporium cladosporioides* MTT23 and *Penicillium citrinum* MTT26. Contreras et al*.*^116^ noted the cytotoxic effects of methylene blue dye on microorganisms at increasing concentrations which explained why the highest rate of removal was obtained at the lowest methylene blue concentration.

Dangerous aromatic compounds can be found in discharged wastewater produced by a range of industries including petroleum refining, coal, plastics, resins, textiles, paper and pulp, iron and steel manufacturing units, and agriculture. Phenols and their derivative compounds are harmful to living organisms even at very low concentrations and are, therefore, considered to be priority pollutants. The methods used to detoxify such compounds are associated with high implementation costs, very low efficiency of removal as a result of incomplete detoxification mechanisms, production of hazardous by-products and are only applicable to a narrow range of pollutant concentrations. This study identified novel fungi that have not been reported previously in the literature, as capable of utilizing crude oil, producing biosurfactants and serving as biocatalysts with a broad potential application to several industries. Cumulatively, these results indicate that MVs can be a unique and valuable source of veritable indigenous fungi that can provide superior performance than commercial/foreign counterparts in bioremediation technologies and remediation strategies that remain site-specific. However, subsequent studies should be performed. These studies should address degradation and genetic studies to identify more accurately the potential and biochemistry involved in remediation.

## Methods

### Site information and sampling

**Figure 5:**
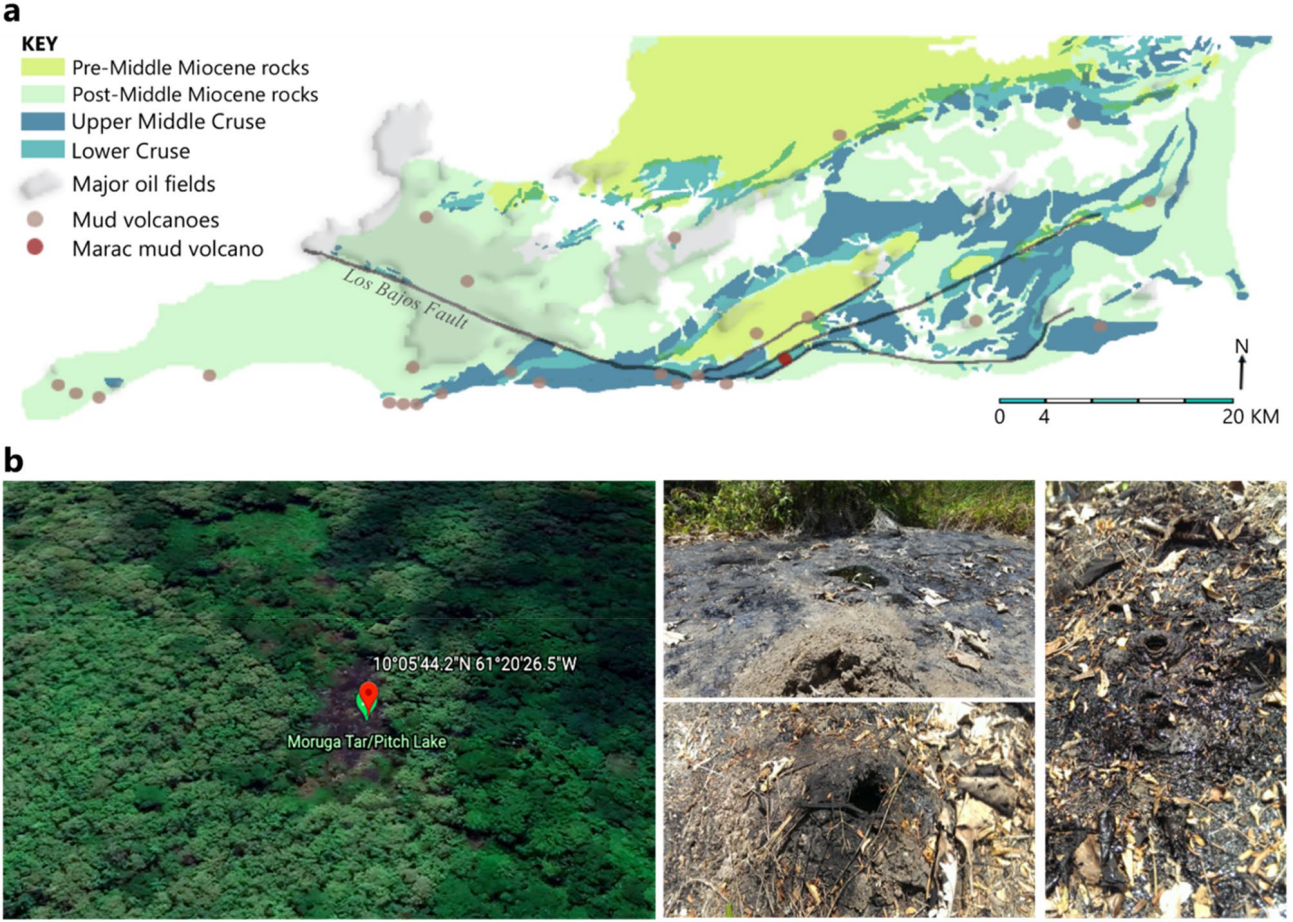
Mud volcanoes (MVs) in Southern Trinidad. (**a**) Location of Marac in Southern Trinidad. Stratigraphically, Marac is located in the Miocene-aged Cruse and Karamat Formation. Geological formations are redrawn from Archie^123^, Vincent^124^ and Soto et al*.*^125^. (Adobe Photoshop 2021; 22.3.1 Release; Elsevier License No. 5132081500912). (**b**) Satellite image of Marac sampling site and active MVs.

The investigated MV is situated in Marac*,* La Lune, Moruga in South Trinidad (10°05′44.2″N, 61°20′26.5″W) (Fig. 5). An extensive description of MV features can be found as a Supplementary Note online. Briefly, surrounded by a semi-evergreen forest, Marac contains a tassik (a round clearing in the forest without vegetation due to an abundance of clay) where oil reservoirs in the form of liquid asphalt and fractures in these asphalt crusts are presented as MVs (Fig. 5). Expellant from MVs is composed of sediment, saline water, liquid hydrocarbons, gases (mainly methane) and minor oil scum^36,121,122^. Recent representative geochemical signatures of MVs in Trinidad revealed mud-systems rich in carbon, nitrogen and sulphur close to the signature of oxidized asphalts and analysis of the water showed an increase in oxygen-carbon ratio with an almost unchanged hydrocarbon deficit. Each MV had a uniquely specific chemical diversity^7^.

Our survey of Marac’s MVs indicated varied levels of seep activity and it was not uniformly stratified. This comes because of cyclic phases of activity^122^. Observed were areas of hardened and softer asphaltic surfaces where humans or animals could sink into, MVs slowly ‘bubbling’ with liquid hydrocarbons, MVs expelling more mud than others as evident by the mounds of mud around the vent and dried up MVs. Despite being in the same oil-bearing area, the variation in activity, source and depth of oil implies the presence of putative complex microbial communities. We noted human disturbance at the site (it is a known hunting location). The ambient temperature was 37 °C, asphaltic temperature was 47 °C, salinity was 3.83 ppt and the pH of the asphalt was 6.72.

MV mud- and oil- derived sediments were collected from four geographically separated locations within the tassik (Table 1). Samples were collected at a depth of 10 cm into the MV subsurface using a stainless steel shovel (500 g per sample). Debris (e.g. pebbles, leaves and twigs) were removed from the samples prior to placing into sterile Whirl–Pak bags which were placed on ice for immediate transportation to the laboratory and stored at 4 °C until processing within 24 h. 

### Isolation of culturable fungi

For isolation of fungi, 1 g of each sample was suspended in 9 mL sterile phosphate buffer pH 7.2 (KH_2_PO_4_ 68 g/L), vortexed for 1 min and serially diluted from 10^–1^ to 10^–5^. An aliquot of 100 μL of each serial dilution was spread over Potato dextrose agar (PDA) and Marine agar (MA) plates supplemented with streptomycin and tetracycline (50 mg/L each). The MVs expel saline water (lower salinity than seawater) with high levels of exchangeable sodium^122^. Therefore, oil-amended PDA supplemented with sodium chloride (1% m/v) and MA were used to simulate the major mineral composition of saline water.

Samples were plated in duplicate under aseptic conditions and incubated for 5 days at 25 °C in the dark. Morphologically different fungal colonies were selected and sub-cultured on PDA and MA to obtain axenic isolates. These were considered unique colonies that would be screened for their oil-degrading capabilities. By performing isolation under these conditions, no competitive interactions occur allowing for a wider number of species to be isolated including rare species in vitro.

### Enrichment for isolation of crude oil degraders

Enrichment was performed to exclusively select for pioneer crude oil-utilizers. For fungal isolation, samples of the MV mud- and oil-derived sediments (10 g per sample) were inoculated in Potato dextrose broth (PDB, 500 mL) and Marine broth (MB, 500 mL)^126^ containing crude oil (2% v/v; CARIRI Laboratories, St. Augustine, Trinidad and Tobago) as the carbon source for enrichment and incubated at 37 °C at 200 rpm for 10 days. After 10 days, inoculum (1 mL) was added to fresh media (100 mL) and incubated again under similar conditions for another 10 days to decrease unwanted microbial load. After incubation, culture media (1 mL) was used for serial dilution in sterile phosphate buffer (pH 7.2) followed by spreading 100 μL from 10^–1^ to 10^–5^ diluted culture on PDA and MA plates and incubated at 25 °C in the dark for 6 days. Morphologically different fungal colonies were then selected and subcultured on PDA and MA to obtain axenic isolates. These were considered as unique colonies that would be screened to further investigate their oil-degrading capabilities.

### Growth bioassays on crude oil

To isolate culturable fungal degraders, growth bioassays on crude oil enriched media were conducted. Plates were inoculated with a 4 mm^3^ block from the advancing edge an actively growing axenic colony. Duplicate assays were carried out under aseptic conditions. Cultures were incubated for 5 days at 25 °C in the dark after which time their growth rate and oil utilization capability was characterized. The crude oil bioassays revealed a unique yeast-bacteria co-culture that showed exceptional oil-degrading potential. As such it was included in the study despite the intended exclusive isolation of fungi (filamentous). Growth rates and performance of all isolates were screened on 2% crude oil enriched PDA and MA plates. Based on their performance on the 2% media, isolates were then grown on the media with superior growth, PDA or MA, amended with 4% and 6% crude oil.

### Oil-tolerance of axenic fungi

Hydrocarbonoclastic isolates obtained from the growth bioassay were characterized and selected for molecular identification. Criteria included: (i) zone of clearance of oil around and/or on the reverse of the colony, (ii) oil droplets around and/or on the reverse of the colony and (iii) high growth rates (diameter, mm/day) of colonies. The top-performing isolates selected had the highest growth rate (≥ 3 mm/day) on the highest concentration of oil (6% v/v). As an additional screen for the top isolates (excluding the yeast-bacterium co-culture isolate), Bushnell-Haas agar (BHA) composed per litre of: MgSO_4_ (0.2 g), CaCl_2_ (0.02 g), KH_2_PO_4_ (1.0 g) K_2_HPO_4_ (1.0 g), NH_4_NO_3_ (1.0 g), FeCl_3_ (0.05 g) and agar (20.0 g) was supplemented with streptomycin and tetracycline (50 mg/L each) and crude oil (2% v/v) as the sole carbon source. The selected isolates were maintained on PDA plates and in 1.5 mL centrifuge tubes in sterile distilled water at 4 °C for short term storage and in 15% glycerol at − 20 °C for long term storage.

### DNA extraction, PCR and sequencing

Hydrocarbonoclastic fungal isolates were grown in PDB supplemented with streptomycin and tetracycline (50 mg/L each) at 25 °C in the dark for 5 days. The mixture was centrifuged for 10 min (12,000*g*, 25 °C), the supernatant was discarded and the mycelia was used to extract total genomic DNA (gDNA) using the MoBio PowerSoil DNA extraction kit (Mo-Bio Laboratories, Carlsbad CA, USA) according to the manufacturer’s instructions. Identification was based on partial sequence comparisons of the internally transcribed spacer (ITS) region (expected PCR product size ~ 650 bp), ITS1-5.8S-ITS2 rDNA array (ITS4/5 primers)^127^. Due to the possibility of incorrect identifications and/or incomplete ITS sequences deposited in the National Centre for Biotechnology Information (NCBI) GenBank, additional approved markers for molecular identification through sequence comparisons of different housekeeping genes are required for accurate species identification, i.e. the β-tubulin (βTUB) gene for *Aspergillus* spp.^128^ and the translation elongation factor 1α (TEF1α) for *Fusarium* sp.^129–131^. Currently, there is no consensus about these supplementary barcodes, since for many taxa, they are genus-dependent.

DNA analysis was also performed to identify the bacterium in co-culture with the yeast. The isolate was grown on Nutrient agar (NA) in the dark for 24 h. Subsequently, the plate was flooded using 500–700 μL of TE buffer pH 8 (10 mM Tris HCl, 1 mM EDTA) and the wash collected and transferred to a 1.5 mL centrifuge tube where 100 μL of lysozyme and proteinase K (50 mg/L each) were added. The samples were incubated at 37 °C for 2 h in a water bath with occasional mixing by inversion. Immediately after incubation, the entire sample content was transferred to a Maxwell 16 Cell DNA Purification kit (Promega, Madison, WI, USA) and gDNA was extracted according to the manufacturer’s protocol.

The PCR mixture (final volume 25 μL) for each reaction included 12.5 μL of GoTaq Green Master Mix (Promega, USA), 0.5 μL (10 μM) of forward and reverse primers (Integrated DNA Technologies, USA), 6.5 μL of Nuclease-Free water (Promega, USA) and 5 μL of DNA template (gDNA dilution 1:4). PCR reaction conditions for fungi included an initial denaturation at 94 °C for 5 min followed by 35 cycles of 94 °C for 1 min, 55 °C for 1 min, 72 °C for 1 min, ending with a final extension at 72 °C for 5 min and for the bacterium, an initial denaturation at 96 °C for 5 min, followed by 33 cycles of 95 °C for 30 s, 55 °C for 30 s, 72 °C for 2 min, ending with a final extension at 72 °C for 2 min. PCR reactions were carried out on Thermal Cycler 2720 (Thermo Scientific, USA). PCR products were examined on 1.5% agarose gels (data not shown) and the amplicons were sent for purification and sequencing (MCLAB, San Francisco, CA, USA).

### Bioinformatic and cluster analyses

Sequences of each amplified gene region were compared to sequences deposited in the NCBI GenBank database using the Basic Local Alignment Search Tool: Nucleotide (BLASTn)^132^. Confirmation of sequence identities was then carried out based on comparative BLASTn results of β-TUB and TEF1α gene regions where applicable. Reference sequences based on the ITS gene were mined and included in the final dataset and are available in Supplementary Table S3. Sequence alignments were performed in MAFFT^133^ (https://www.ebi.ac.uk/Tools/msa/mafft/) and edited in BioEdit^134^. Unrooted phylogenetic trees for the ITS sequences were constructed in MEGA6^135^ based on Maximum Likelihood estimations using the best predicted model of nucleotide substitution after 1000 replicates.

### Statistical analysis and software

Minitab software (version 19) was used to perform analysis of growth rates on oil-supplemented media using Fisher’s Pairwise comparisons and significant differences were calculated using one-way analysis of variance (ANOVA). RStudio software (version 1.3.1093)^136^ statistical programming environment was used to perform analyses. α-diversity metrics including the Shannon diversity index (H), Simpson diversity index (D_1_), absolute richness (S)^137^ and Chao 1 richness estimator^138^ were generated using the package ‘BiodiversityR’ and absolute dominance and Simpson’s dominance (D_2_) indices^139^ using the package ‘microbiome’. Abundance statistics and the species abundance pattern using Rank-abundance dominance (RAD) analysis was performed using the package ‘BiodiversityR’ after the best-fit model was selected based Akaike information criterion (AIC)^140^. Figures were produced using the packages ‘ggplot2’, ‘scales’, and ‘reshape’. ChemBioDraw (level: ultra, version 12.0) was used to draw all chemical structures of substrates used in the oxidoreductase experiment.

### Biochemical assays

#### Biosurfactant assay

The top-performing fungal isolates were screened (*N* = 18) for biosurfactant production using the oil-spreading assay as a sensitive and rapid method that can detect low activity and quantities of biosurfactant with no purification required^86,141,142^. Crude biosurfactant for the assay was prepared using 25 mL BHB, composed per litre of: MgSO_4_ (0.2 g), CaCl_2_ (0.02 g), KH_2_PO_4_ (1.0 g) K_2_HPO_4_ (1.0 g), NH_4_NO_3_ (1.0 g) and FeCl_3_ (0.05 g), sterilized crude oil (2% v/v) as the sole carbon source, a 4 mm^3^ inoculum from the advancing colony edge of an actively growing axenic culture and incubation at 25 °C in the dark with shaking (120 rpm) for 7 days. As a control, uninoculated flasks were also prepared. Assays were prepared in triplicate and the crude supernatant used in the oil-spreading assay.

A Petri dish was filled with 20 mL of distilled water, colored with yellow food coloring for better visibility, and 100 μL of paraffin oil was placed carefully at the center to form an oil layer on the surface. 20 μL of crude supernatant was pipetted onto the center of the paraffin oil layer. The presence of biosurfactants is confirmed by oil displacement activity where there is a clearing zone like a halo forming. A negative control with uninoculated BHB was also evaluated. Assays were performed in triplicate. A visual of the oil-spreading assay can be viewed in Supplementary Video 1 and 2 and the control in Supplementary Video 3 and 4 online.

### Secreted oxidoreductase determination

The ability of selected crude oil-utilizers to metabolize different substrates through the action of extracellularly secreted oxidoreductases was assessed. In a preliminary experiment, optimum conditions for enzyme activity were determined i.e. pH, temperature, buffer, reaction time and substrate concentration.

Top-performing fungal isolates (*N* = 18) were grown in BHB in the dark for 7 days at 35 °C at 200 rpm. Mycelia was removed by centrifugation (10 min, 12,000*g*, rt) and the specific activity of oxidoreductases present in the crude culture supernatant was assessed for different substrates. Assays were conducted spectrophotometrically and the mean change in absorbance over time was calculated for each substrate. Oxidoreductase activity was assayed using a range of substrates (5 mM)^143^ including: (i) gallic acid, (ii) catechol, (iii) hydroquinone, (iv) resorcinol, (v) orcinol, (vi) ABTS, (vii) tannic acid and (viii) methylene blue. To confirm the fungal origin of oxidoreductase activity detected, negative controls were solely BHB without any fungal inoculum. The enzyme assays were carried out simultaneously with the negative controls in duplicate.

## Supplementary Information

Supplementary Information 1.

Supplementary Information 2.

## Data Availability

The datasets generated during and/or analysed during the current study are available from the corresponding author upon reasonable request. Nucleotide sequences of each strain are available in the NCBI GenBank database under the accession numbers: MZ143960-MZ143989.
