# Supplementary material for: Biodiversity and biocatalyst activity of culturable hydrocarbonoclastic fungi isolated from Marac–Moruga mud volcano in South Trinidad
Source: Sci Rep. 2021 Sep 30;11:19466. doi: 10.1038/s41598-021-98979-6 (PMC8484666; doi:10.1038/s41598-021-98979-6)
Supplement: Supplementary file 2 — Supplementary Information 2. [file 41598_2021_98979_MOESM2_ESM.docx]

**Biodiversity and biocatalyst activity of culturable hydrocarbonoclastic fungi isolated from Marac-Moruga mud volcano in South Trinidad**

**Amanda C. Ramdass^1^, and Sephra N. Rampersad^1*^**

^1^ The University of the West Indies, Faculty of Science and Technology, Department of Life Sciences, Biochemistry Research Laboratory (Rm216), St. Augustine, Trinidad and Tobago - West Indies

*****sephra.rampersad@sta.uwi.edu

**Supplementary Material**

**Supplementary Note**

**Marac mud volcano site information.** The Marac tassik, is the “tip of the iceberg” in relation to the subsurface volume of mud and varies in shape as a result of the frequent venting of underlying mud. Clay cones of protruding mud can form from a few centimetres up to several metres [1,2]. In South Trinidad, asphaltic deposits are concentrated in the Pilo-Pleistocene Formations and the associated MVs are formed as fractures along an active belt of subsurface sediment mobilization processes that span several hundred kilometers starting from the Barbados tectonic wedge to the thrust belt of Northern Venezuela [3-5]. In Marac, the reservoir deposits are likely associated with the Miocene-aged Cruse Formation and asphaltic paleontological records have shown that Miocene black shale is the source material of these MVs [2,6].

Primarily, the expellant is composed of sediment (mostly clay and clasts), saline water (pH 7-8.2, with high levels of exchangeable sodium with a salinity less that seawater), liquid hydrocarbons (fluids reflect the present state of hydrocarbons produced at depth), gases (purely thermogenic and mainly methane) and minor oil scum [2,5,7]. Fluids expelled reflect the present state of hydrocarbons at depth but are not necessarily a representation of trapped hydrocarbons at depth [8]. Throughout evolutionary time, high temperature fluid-rock interactions changed the original oceanic chemical composition [7]. To gain a more up-to-date and representative geochemical signature of MVs in Trinidad, a study by Schulze-Makuch*, et al.* [9] based on Fourier-Transform Ion-Cyclotron-Resonance Mass Spectrometry (FTICR-MS) showed that the mud-systems were rich in carbon, nitrogen, and sulphur which were close to the signature of oxidized asphalts whereas analysis of the water showed an increase in oxygen-carbon ratio (due to oxidation reactions) with an almost unchanged hydrocarbon deficit. When compared, each MV had a uniquely specific chemical diversity [9]. The volcanoes at this site are not strongly eruptive and over time, vent locations can change. Temperatures at this site can vary because of the temperature dynamics of two-phase flow of gas and mud through the MV channels [4].

**References**

1. Beard, J. S. *The Natural Vegetation of Trinidad* (Clarendon Press, 1946).

2. Higgins, G. & Saunders, J. Mud volcanoes-their nature and origin, contribution to the geology and paleobiology of the Carribean adjacent areas. *Verhand. der Natur. Geschechaft zu Bazel.* **84**, 121-152 (1974).

3. Castrec-Rouelle, M., Bourlès, D. L., Boulègue, J. & Dia, A. N. Beryllium geochemistry constraints on the hydraulic behavior of mud volcanoes: the Trinidad island case. *Earth Planet. Sci. Lett.* **203**, 957-966; 10.1016/S0012-821X(02)00922-6 (2002).

4. Deville, E. *et al.* The origin and processes of mud volcanism: new insights from Trinidad. *Geol. Soc. Spec. Publ.* **216**, 475; 10.1144/GSL.SP.2003.216.01.31 (2003).

5. Deville, E. & Guerlais, S. H. Cyclic activity of mud volcanoes: evidences from Trinidad (SE Caribbean). *Mar. Pet. Geol.* **26**, 1681-1691; 10.1016/j.marpetgeo.2009.03.002 (2009).

6. Aslan, A. *et al.* Mud volcanoes of the Orinoco Delta, Eastern Venezuela. *Geomorphology* **41**, 323-336; 10.1016/S0169-555X(01)00065-4 (2001).

7. Dia, A. N., Castrec-Rouelle, M., Boulègue, J. & Comeau, P. Trinidad mud volcanoes: where do the expelled fluids come from? *Geochim. Cosmochim. Acta* **63**, 1023-1038; 10.1016/S0016-7037(98)00309-3 (1999).

8. Battani, A., Prinzhofer, A., Deville, E. & Ballentine, C. J. *Shale tectonics: AAPG memoir 93* (ed. Wood, L. J.) 225–238 (American Association of Petroleum Geologists, 2010).

9. Schulze-Makuch, D. *et al.* A chemical and microbial characterization of selected mud volcanoes in Trinidad reveals pathogens introduced by surface water and rain water. *Sci. Total Environ.* **707**, 136087; 10.1016/j.scitotenv.2019.136087 (2020).

**Supplementary Figures**

**
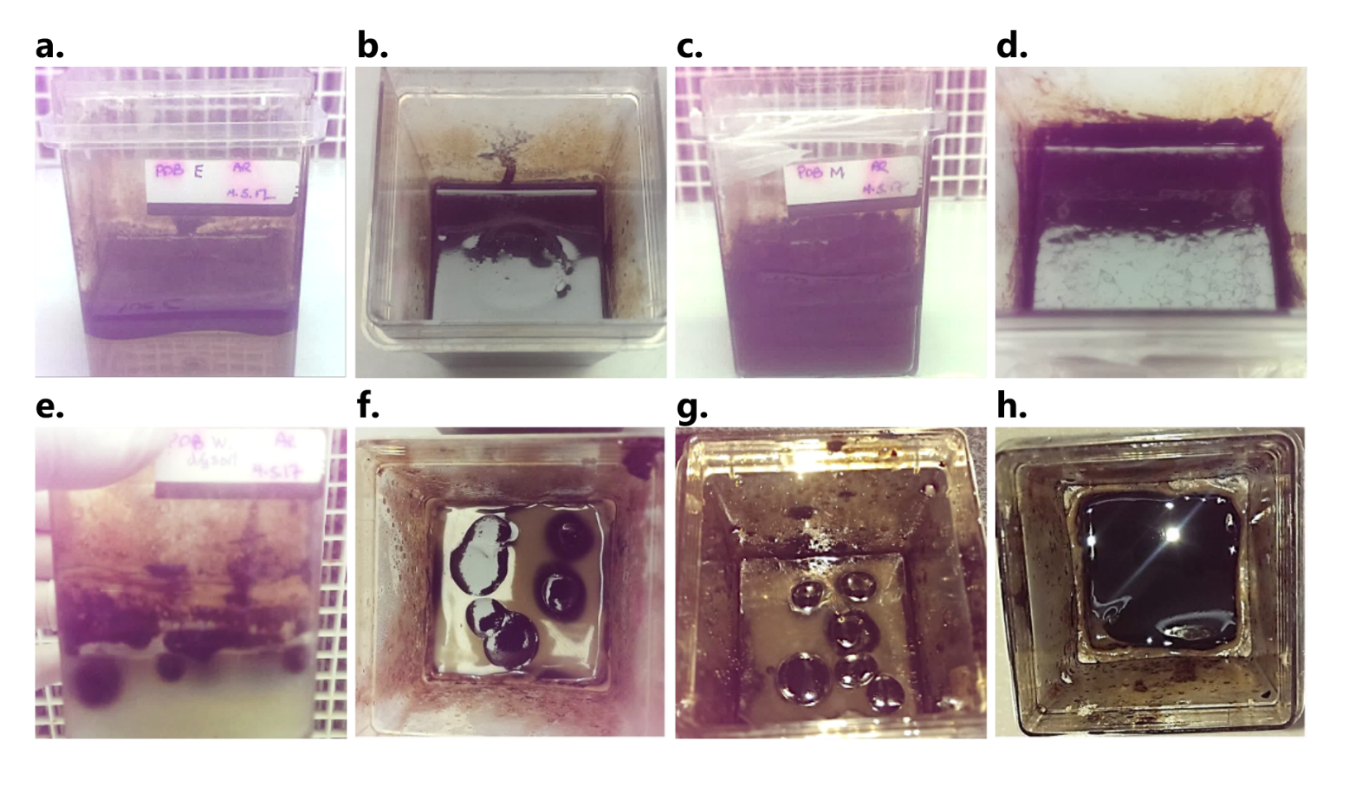
**

**Supplementary Figure S1.** Spherical oil globules of fungal cultures from soil samples grown in PDB. Negative controls consisted of (i) crude oil only in PDB, and (ii) Tween 20 added to (i).


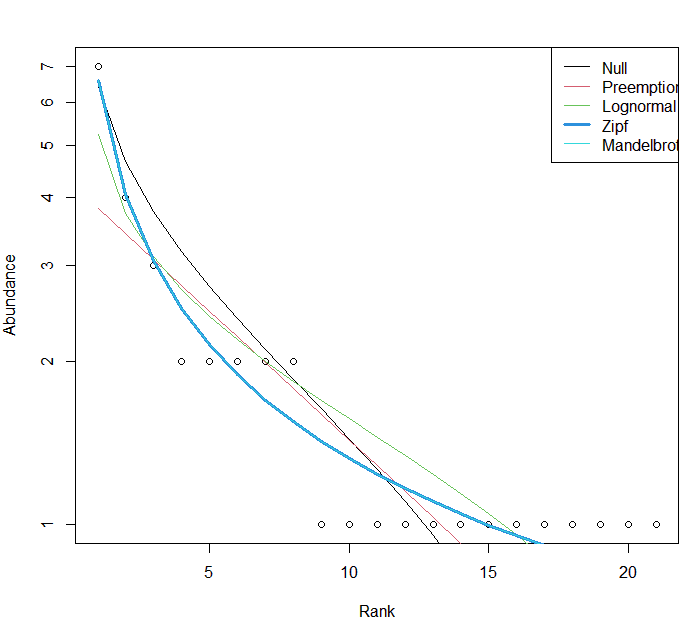


**Supplementary Figure S2.** Rank-abundance dominance (RAD) plots for genera distribution of fungi. The best model is represented by the bolded line - Zipf model. Null represents the MacArthur model; Preemption represents the geometric series of Motomura Model; Lognormal represents the Lognormal distribution of Preston; Zipf represents the Zipf Model; Mandelbrot represents the Zipf-Mandelbrot Model [1]. (RStudio software v1.3.1093).

**Supplementary Tables**

**Supplementary Table S1.** Quantitative and qualitative characteristics of the 37 hydrocarbonoclastic fungal isolates on oil-amended media. Growth rate is given as diameter/mm/day. *Represents an overgrown culture and no measurement was taken. x represents an isolate that was not screened on this media and thus no observation of performance was recorded. Performance was recorded as presence or absence (+/-) for a zone of clearance of oil around or on the reverse of the colony (Z) or droplets of oil around or on reverse of the colony (D), (Z/D). The top 19 hydrocarbonoclastic isolates in group A with the highest oil-tolerance are highlighted in blue where growth rate on 6% crude oil enriched media was more than or equal to 3 mm/day (apart from isolate 37); the isolate with the highest growth rate on a given media is highlighted in purple; the isolate with the lowest growth rate on a given media is highlighted in orange.

| **Isolate** | **2% PDA** | **Z/D** | **2% MA** | **Z/D** | **4% PDA** | **Z/D** | **4% MA** | **Z/D** | **6% PDA** | **Z/D** | **6% MA** | **Z/D** | **2% BHA** | **Z/D** |
| --- | --- | --- | --- | --- | --- | --- | --- | --- | --- | --- | --- | --- | --- | --- |
| MTT1 | * | +/- | 4.82 | +/- | 3.61 | +/- | 4.73 | +/+ | 3.95 | +/- | 4.73 | -/+ | 7.01 | -/- |
| MTT2 | * | +/- | * | +/+ | 6.64 | +/+ | 9.77 | +/+ | 6.07 | +/- | 10.00 | +/+ | 11.24 | -/- |
| MTT3 | * | +/- | * | +/+ | * | -/- | 4.80 | -/- | * | -/- | 4.23 | -/+ | * | -/- |
| MTT4 | * | +/- | 5.68 | +/+ | 4.70 | +/+ | 9.55 | +/- | 4.86 | +/- | 9.09 | -/+ | 12.70 | -/- |
| MTT5 | * | +/- | 7.82 | +/- | 5.34 | +/- | 6.45 | +/+ | 4.95 | +/- | 6.23 | +/+ | 7.50 | -/- |
| MTT6 | * | +/+ | 0.00 | -/- | 6.07 | +/- | 0.00 | -/- | 6.41 | +/+ | 0.00 | -/- | 0.00 | -/- |
| MTT7 | * | -/- | 7.00 | +/- | * | -/- | 5.18 | +/+ | * | -/- | 5.43 | -/+ | * | -/- |
| MTT8 | 5.23 | +/- | 3.86 | -/- | 3.50 | +/- | 4.82 | +/+ | 3.73 | +/- | 5.16 | -/+ | 7.20 | -/- |
| MTT9 | 1.27 | +/- | 0.66 | +/- | * | -/- | 0.64 | -/+ | 0.64 | -/- | 0.52 | -/+ | x | x |
| MTT10 | 2.00 | +/- | 1.52 | +/- | * | -/- | 1.07 | -/+ | 0.95 | -/+ | 1.27 | -/+ | x | x |
| MTT11 | 2.05 | +/- | 1.55 | +/- | * | -/- | 1.07 | -/+ | 2.14 | -/+ | 1.18 | -/+ | x | x |
| MTT12 | 5.25 | +/- | 3.36 | +/- | 1.77 | +/- | 2.73 | -/+ | 2.36 | -/- | 2.86 | +/- | x | x |
| MTT13 | 3.34 | +/- | 3.02 | +/- | 2.93 | -/- | 1.32 | -/- | 3.30 | +/- | 1.57 | -/- | 5.53 | +/- |
| MTT14 | * | -/- | 5.66 | +/- | * | +/- | 4.66 | +/+ | * | -/- | 5.18 | +/- | * | -/- |
| MTT15 | 2.91 | +/- | 4.41 | +/- | 2.50 | -/- | 3.55 | +/- | 2.82 | -/- | 3.02 | -/- | 4.13 | +/- |
| MTT16 | 4.00 | +/- | 2.26 | +/- | 3.68 | +/- | x | x | 3.64 | -/- | x | x | 4.23 | -/- |
| MTT17 | 7.27 | +/- | 4.41 | +/- | 6.23 | +/- | x | x | 5.77 | -/- | x | x | 4.26 | -/- |
| MTT18 | 3.00 | +/- | 2.00 | +/- | 2.45 | +/- | x | x | 2.73 | -/- | x | x | x | x |
| MTT19 | 1.52 | +/- | 1.16 | +/- | 1.32 | +/- | x | x | 1.41 | +/+ | x | x | x | x |
| MTT20 | 2.16 | +/- | 1.00 | +/- | 1.82 | +/- | x | x | 1.86 | +/+ | x | x | x | x |
| MTT21 | 1.36 | +/- | 0.91 | +/- | 1.34 | +/- | x | x | 1.41 | +/- | x | x | x | x |
| MTT22 | 1.98 | +/- | 0.77 | +/- | 1.27 | +/- | x | x | 1.57 | +/- | x | x | x | x |
| MTT23 | 2.20 | +/- | 1.32 | +/- | 1.89 | +/- | x | x | 3.00 | +/- | x | x | 3.13 | +/- |
| MTT24 | 3.11 | +/- | 1.75 | +/- | 2.25 | +/- | x | x | 3.02 | +/- | x | x | 5.24 | -/- |
| MTT25 | 3.41 | +/- | 2.73 | +/- | 3.27 | +/- | x | x | 3.68 | +/- | x | x | 4.69 | -/- |
| MTT26 | 2.80 | +/- | 2.55 | +/- | 2.57 | +/- | x | x | 3.82 | +/- | x | x | 3.79 | -/- |
| MTT27 | 1.57 | +/- | 1.18 | +/- | 1.11 | +/- | x | x | 1.39 | +/- | x | x | x | x |
| MTT28 | 5.68 | +/- | 4.30 | +/- | 6.36 | +/- | x | x | 5.61 | +/+ | x | x | 6.24 | -/- |
| MTT29 | 2.59 | +/+ | 1.98 | +/- | 2.02 | +/- | x | x | 2.64 | +/+ | x | x | x | x |
| MTT30 | 3.18 | +/+ | 2.27 | +/- | 2.91 | +/- | x | x | 2.18 | +/+ | x | x | x | x |
| MTT31 | 1.98 | +/- | 1.39 | +/- | 2.25 | +/- | x | x | 1.57 | +/+ | x | x | x | x |
| MTT32 | 3.95 | +/- | 1.91 | +/- | 3.14 | +/- | x | x | 2.55 | +/+ | x | x | x | x |
| MTT33 | 3.50 | +/- | 1.91 | +/- | x | x | 2.95 | +/+ | x | x | 2.23 | -/+ | x | x |
| MTT34 | 1.57 | +/- | 1.91 | +/- | x | x | 3.25 | +/- | x | x | 2.27 | +/+ | x | x |
| MT35 | 2.28 | -/- | x | x | x | x | 1.09 | -/- | x | x | 0.68 | -/+ | x | x |
| MTT36 | * | -/- | * | -/- | * | -/- | * | -/- | * | -/- | * | -/- | x | x |
| MTT37 | 2.41 | +/+ | 0.82 | +/+ | 1.48 | +/+ | x | x | 1.77 | +/+ | x | x | x | x |

**Supplementary Table S2.** Descriptive statistics and Fisher pairwise comparisons for growth on media supplemented with different concentrations of crude oil. *Grouping information using the Fisher LSD Method and 95% confidence where means that do not share the same letter are significantly different.

| **Media** | **N** | **Mean** | **SE Mean** | **St Dev** | **Minimum** | **Maximum** | **Grouping*** |
| --- | --- | --- | --- | --- | --- | --- | --- |
| 2% MA | 33 | 2.663 | 0.333 | 1.913 | 0.000 | 7.820 | A |
| 2% PDA | 28 | 2.985 | 0.273 | 1.444 | 1.270 | 7.270 | A |
| 4% MA | 18 | 3.757 | 0.667 | 2.832 | 0.000 | 9.770 | A |
| 4% PDA | 27 | 3.127 | 0.327 | 1.698 | 1.110 | 6.640 | A |
| 6% MA | 18 | 3.647 | 0.674 | 2.858 | 0.000 | 10.000 | A |
| 6% PDA | 30 | 3.060 | 0.288 | 1.579 | 0.640 | 6.410 | A |

**Supplementary Table S3.** Identification of fungi, yeast and bacterium of the oil-tolerant strains. GenBank accession information is provided where two references are provided per species except in cases where only one reference was available. The placement of fungi into respective taxonomic levels was aided by the National Center for Biotechnology Information (NCBI) Taxonomy browser (https://www.ncbi.nlm.nih.gov/Taxonomy/Browser/wwwtax.cgi).

| **Isolate** | **Species** | | **Phylum** | **Class** | **QC%/ID%** | **Country** | **Host/Source** | **Accession** |
| --- | --- | --- | --- | --- | --- | --- | --- | --- |
| **Fungi** |  | |  |  |  |  |  |  |
| MTT1 | *Aspergillus ochraceopetaliformis/flocculosus* | | *Ascomycota* | *Eurotiomycetes* | 100/100 | Singapore | Soil | KR296859 |
|  |  | |  |  |  | Thailand | House dust | KP329610 |
| MTT2 | *Fusarium equiseti/incarnatum* | | *Ascomycota* | *Sordariomycetes* | 99/99.38 | Malaysia | Leachate contaminated soil | MK209007 |
|  |  | |  |  |  | China | Tomato | MK212925 |
| MTT3 | *Neoscytalidium hyalinum/dimidiatum* | | *Ascomycota* | *Dothideomycetes* | 100/100 | France |  | MH863577 |
|  |  | |  |  |  | Gabon |  | MH863572 |
| MTT4 | *Fusarium equiseti/incarnatum* | | *Ascomycota* | *Sordariomycetes* | 100/100 |  |  |  |
| MTT5 | *Aspergillus flavus* | | *Ascomycota* | *Eurotiomycetes* | 100/99.82 | India | Muccuna pruriens | MN238861 |
|  |  | |  |  |  | China |  | MT584825 |
| MTT6 | *Aspergillus flavus* | | *Ascomycota* | *Eurotiomycetes* | 99/80.43 (TEF98/90.65) |  |  |  |
| MTT7 | *Neoscytalidium hyalinum/dimidiatum* | | *Ascomycota* | *Dothideomycetes* | 100/97.79 |  |  |  |
| MTT8 | *Alternaria porri/tenuissima/destruens* | | *Ascomycota* | *Dothideomycetes* | 100/100 | India |  | MT554514 |
| MTT9 | *Cladosporium dominicanum/sphaerospermum* | | *Ascomycota* | *Dothideomycetes* | 100/99.81 | China | Mangrove sediments | KY827344 |
|  |  | |  |  |  | USA | Outside air sample | MF472967 |
| MTT10 | *Cladosporium cladosporioides/tenuissimum/colombiae/oxysporum* | | *Ascomycota* | *Dothideomycetes* | 100/100 | Antarctica | Soil | MT367253 |
|  |  | |  |  |  | China | Sea water | MK732117 |
| MTT11 | *Cladosporium cladosporioides/tenuissimum/colombiae/oxysporum* | | *Ascomycota* | *Dothideomycetes* | 99/100 |  |  |  |
| MTT12 | *Stagonosporopsis cucurbitacearum* | | *Ascomycota* | *Dothideomycetes* | 99/100 | Taiwan | Sechium edule | AB714986 |
|  |  | |  |  |  | Australia | Nicotiana simulans (root) | KU059901 |
| MTT13 | *Phoma* sp. | | *Ascomycota* | *Dothideomycetes* | 100/100 | China | Praxelis clematidea | JN709462 |
|  |  | |  |  |  | India | Infected pseudostem of small cardamom | MN962956 |
| MTT14 | *Neoscytalidium hyalinum/dimidiatum* | | *Ascomycota* | *Dothideomycetes* | 100/99.10 |  |  |  |
| MTT15 | *Ascomycete* sp. | | *Ascomycota* | *Dothideomycetes* | 100/98.34 | Thailand |  | EF632079 |
| MTT16 | *Montagnula scabiosae* | | *Ascomycota* | *Dothideomycetes* | 51/82.26 | China | Fluorocarbon coating | MW081285 |
|  |  | |  |  | 51/81.99 | Sri Lanka | Pyrenocarp (stem bark) | MK224450 |
| MTT17 | *Montagnula opulenta* | | *Ascomycota* | *Dothideomycetes* | 100/97.64 | USA |  | LT796834 |
|  |  | |  |  |  | Mauritius | Marine sponge | MW187736 |
| MTT18 | *Allophoma minor* | | *Ascomycota* | *Dothideomycetes* | 99/100 | Indonesia | Culture from holotype of Allophoma minor | MH861501 |
|  |  | |  |  | 96/100 | Netherlands | Culture from holotype of Allophoma minor | NR_135989 |
| MTT19 | *Aspergillus stromatoides* | | *Ascomycota* | *Eurotiomycetes* | 99/100 | USA | Culture from lectotype of Aspergillus stromatoides | NR_137454 |
|  |  | |  |  | 99/99.82 | India | Marine Estuary | MN186834 |
| MTT20 | *Aspergillus stromatoides* | | *Ascomycota* | *Eurotiomycetes* | 87/99.34 |  |  |  |
| MTT21 | *Cladosporium cladosporioides/xanthochromaticum/rugulovarians/delicatulum* | | *Ascomycota* | *Dothideomycetes* | 100/99.50 |  |  |  |
| MTT22 | *Cladosporium cladosporioides/xanthochromaticum/delicatulum* | | *Ascomycota* | *Dothideomycetes* | 100/100 |  |  |  |
| MTT23 | *Cladosporium cladosporioides/tenuissimum/colombiae/oxysporum* | | *Ascomycota* | *Dothideomycetes* | 100/100 |  |  |  |
| MTT24 | *Penicillium paxilli* | | *Ascomycota* | *Eurotiomycetes* | 100/100 | Brazil | Digestive tract of Phylloicus elektoros | MK120566 |
|  |  | |  |  |  | Panama | Culture from neotype of Penicillium paxilli | MH856391 |
| MTT25 | *Penicillium citrinum* | | *Ascomycota* | *Eurotiomycetes* | 98/97.17 |  |  |  |
| MTT26 | *Penicillium citrinum* | | *Ascomycota* | *Eurotiomycetes* | 100/100 | Korea | Air | MT582768 |
|  |  | |  |  |  | China | Rosy rice vinegar solid mash | MT558921 |
| MTT27 | *Cladosporium cladosporioides/xanthochromaticum/delicatulum/colombiae* | | *Ascomycota* | *Dothideomycetes* | 100/99.80 |  |  |  |
| MTT28 | *Aspergillus niger* | | *Ascomycota* | *Eurotiomycetes* | 100/100 | Egypt | Agriculture soil | MW332264 |
|  |  | |  |  |  | India | Waste water effluent | MT628904 |
| MTT29 | *Penicillium citrinum* | | *Ascomycota* | *Eurotiomycetes* | 100/100 |  |  |  |
| MTT30 | *Penicillium citrinum* | | *Ascomycota* | *Eurotiomycetes* | 66/88.78 |  |  |  |
| MTT31 | *Penicillium oxalicum* | | *Ascomycota* | *Eurotiomycetes* | 100/100 | China | Air | MW077050 |
|  |  | |  |  |  | India | Lake water | MF326633 |
| MTT32 | *Penicillium oxalicum* | | *Ascomycota* | *Eurotiomycetes* | 100/100 |  |  |  |
| MTT33 | *Penicillium chrysogenum* | | *Ascomycota* | *Eurotiomycetes* | 100/100 | USA |  | MH865988 |
|  |  | |  |  |  | Qatar | Inland Sea water | KY781802 |
| MTT34 | *Penicillium chrysogenum/commune/vanluykii* | | *Ascomycota* | *Eurotiomycetes* | 99/95.17 |  |  |  |
| MTT35 | *Cladosporium cladosporioides/tenuissimum/colombiae/oxysporum* | | *Ascomycota* | *Dothideomycetes* | 100/100 |  |  |  |
| MTT36 | *Neurospora crassa/intermedia* | | *Ascomycota* | *Sordariomycetes* | 100/100 | Vietnam | Coastal marine habitat | MT102855 |
|  |  | |  |  |  | China | Spontaneously fermented soybean residue | KX458113 |
| **Yeast** |  | |  |  |  |  |  |  |
| MTT37 | *Momyces parantarcticus=Pseudozyma parantarctica* | | *Basidiomycota* | *Ustilaginomycetes* | 99/100 | Thailand |  | KY104283 |
|  |  | |  |  |  | Taiwan |  | JN544036 |
| **Bacterium** | |  |  |  |  |  |  |  |
| MTT37 | *Janthinobacterium lividum* | | *Proteobacteria* | *Betaproteobacteria* | 100/99.88 | China | Soil | KF583727 |
|  |  | |  |  |  | Mexico | Riverbank soil | KU144683 |

**Supplementary Table S4.** The Akaike information criterion (AIC) values for the species rank abundance distribution models of the fungal community.

| **No. of species** | **Abundance** | **Null** | **Preemption** | **Lognormal** | **Zipf** | **Mandelbrot** |
| --- | --- | --- | --- | --- | --- | --- |
| 21 | 37 | 58.591 | 55.979 | 56.026 | 53.917 | 59.051 |

**Supplementary Table S5.** Ranked abundance of fungal taxonomic levels detected. *Single oil tolerant yeast identified.

| **Taxonomic level** | **Rank** | **Abundance** | **Proportion** |
| --- | --- | --- | --- |
| **Class** |  |  |  |
| *Dothideomycetes* | 1 | 18 | 48.649 |
| *Eurotiomycetes* | 2 | 15 | 40.541 |
| *Sordariomycetes* | 3 | 3 | 8.108 |
| *Ustilaginomycetes** | 4 | 1 | 2.703 |
| **Genus** |  |  |  |
| *Penicillium* | 1 | 9 | 24.324 |
| *Cladosporium* | 2 | 8 | 21.622 |
| *Aspergillus* | 3 | 6 | 16.216 |
| *Neoscytalidium* | 4 | 3 | 8.108 |
| *Fusarium* | 5 | 2 | 5.405 |
| *Montagnula* | 6 | 2 | 5.405 |
| *Alternaria* | 7 | 1 | 2.703 |
| *Stagonosporopsis* | 8 | 1 | 2.703 |
| *Ascomycete* | 9 | 1 | 2.703 |
| *Phoma* | 10 | 1 | 2.703 |
| *Allophoma* | 11 | 1 | 2.703 |
| *Neurospora* | 12 | 1 | 2.703 |
| *Pseudozyma** | 13 | 1 | 2.703 |
| **Species** |  |  |  |
| *Cladosporium cladosporioides* | 1 | 7 | 18.919 |
| *Penicillium citrinum* | 2 | 4 | 10.811 |
| *Neoscytalidium hyalinum* | 3 | 3 | 8.108 |
| *Aspergillus flavus* | 4 | 2 | 5.405 |
| *Fusarium equiseti* | 5 | 2 | 5.405 |
| *Penicillium chrysogenum* | 6 | 2 | 5.405 |
| *Penicillium oxalicum* | 7 | 2 | 5.405 |
| *Aspergillus stromatoides* | 8 | 2 | 5.405 |
| *Cladosporium dominicanum* | 9 | 1 | 2.703 |
| *Ascomycete* sp. | 10 | 1 | 2.703 |
| *Allophoma minor* | 11 | 1 | 2.703 |
| *Phoma* sp. | 12 | 1 | 2.703 |
| *Stagonosporopsis cucurbitacearum* | 13 | 1 | 2.703 |
| *Montagnula opulenta* | 14 | 1 | 2.703 |
| *Montagnula scabiosae* | 15 | 1 | 2.703 |
| *Aspergillus ochraceopetaliformis* | 16 | 1 | 2.703 |
| *Alternaria porri* | 17 | 1 | 2.703 |
| *Aspergillus niger* | 18 | 1 | 2.703 |
| *Penicillium paxilli* | 19 | 1 | 2.703 |
| *Neurospora crassa* | 20 | 1 | 2.703 |
| *Pseudozyma parantarctica** | 21 | 1 | 2.703 |

**Supplementary Table S6.** Substrates screened for the top 18 oil-degrading fungal isolates for extracellular oxidoreductase activity against 8 tested substrates. Substrates are categorized into 5 groups according to chemical group. Activity towards a substrate based on a change of absorbance is given as (+) and no activity as (-). The isolate with the highest activity for a given substrate is highlighted in blue.

|  | **Aromatic carboxylic acid** | **Aromatic alcohols** | | | | **Aromatic azo** | **Polyphenol** | **Thiazine dye** |
| --- | --- | --- | --- | --- | --- | --- | --- | --- |
| **Substrate** | Gallic acid | Catechol | Hydroquinone | Resorcinol | Orcinol | ABTS | Tannic acid | Methylene blue |
| **Absorbance/nm** | 273 | 278 | 278 | 278 | 278 | 420 | 420/500 | 668 |
| **MTT1** | + | + | + | + | + | + | + | **+** |
| **MTT2** | + | - | + | + | + | + | + | + |
| **MTT3** | + | + | + | - | + | + | + | + |
| **MTT4** | **+** | + | + | + | + | + | + | - |
| **MTT5** | + | + | - | + | + | + | - | + |
| **MTT6** | + | + | + | **+** | + | + | + | - |
| **MTT7** | + | + | - | + | + | + | + | + |
| **MTT8** | + | + | **+** | + | + | + | **+** | + |
| **MTT13** | - | + | + | + | + | + | + | + |
| **MTT14** | - | + | - | - | - | + | + | - |
| **MTT15** | + | + | + | + | **+** | + | + | + |
| **MTT16** | - | + | + | + | + | **+** | + | - |
| **MTT17** | - | **+** | + | + | + | + | + | + |
| **MTT23** | - | + | + | + | + | + | - | + |
| **MTT24** | - | + | + | + | + | + | - | - |
| **MTT25** | + | + | + | + | + | + | + | - |
| **MTT26** | - | + | + | + | + | + | + | + |
| **MTT28** | + | + | + | + | + | + | + | - |

**Supplementary Table S7.** Classes of biosurfactants and representative producing microorganisms. Fungi [2-10], yeast [3-8,11-17] and bacteria [4-8,13,15,16,18-31] biosurfactant producers associated with petroleum hydrocarbons are in bold and others are representative producing microorganisms that have a wide range of industrial applications including use in the field of bioremediation. References for “unspecified” fungi [3,32,33], yeast [34,35] and bacteria [30,36-38] mentioned.

| **Class** | **Subclass** | **Microorganisms** |
| --- | --- | --- |
| **Fatty acids** | Caprylic acids | Bacteria*: Pseudomonas*, *Burkholderia* |
|  | Carynomycolic acid | Bacterium: *Corynebacterium lepus* |
|  | Fatty acids (unspecified) | Bacteria: *Acinetobacter* sp., *Arthrobacter paraffineus*, *Capnocytophaga* sp., *Corynebacterium insidibasseosum*, *Corynebacterium lepus*, *Nocardia erythropolis*, *Rhodococcus erythropolis*, *Talaramyces trachyspermus* |
|  | Oleic acid | Yeast: *Issatchenkia orientalis* |
|  | Polysaccharide-fatty acid complex | Yeast: *Candida tropicalis* |
|  | Spiculisporic acid | Fungus: *Penicillium spiculisporum* |
|  | Monoglycerides | Fungus: *Exophiala dermatitidis* SK80 |
| **Flavolipids** |  | Bacterium: *Flavobacterium* sp. |
| **Glycolipids** | Cellobiose lipid (microcin) | Fungi: *Ustilago maydis*, *Ustilago zeae*  Bacterium: *Cryptococcus humicola* |
|  | Flocculosin | Yeast: *Pseudozyma flocculosa* |
|  | Glucose lipids | Bacterium: *Alcanivorax borkumensis* |
|  | Glycolipids (unspecified) | Fungi: *Aspergillus niger*, *Fusarium* sp., *Penicillium citrinum*  Yeast: *Alcanivorax borkumensis*, *Candida antartica*, *Candida apicola*, *Candida batistae*, *Candida bogoriensis*, *Candida bogoriensis*, *Candida bombicola*, *Candida glabrata*, *Candida ishiwadae*, *Candida lipolytica*, *Candida sphaerica*, *Kurtzmanomyces* sp., *Pseudozyma antarctica*, *Pseudozyma aphidis*, *Pseudozyma fusifornata*, *Pseudozyma rugulosa*, *Wickerhamomyces anomalus*  Bacteria: *Bacillus* sp., *Aeromonas* sp., *Brevibacterium* sp., *Ochrobactrum* sp., *Rhodococcus erythropolis*, *Rhodococcus wratislaviensis*, *Acinetobacter* *calcoaceticus*, *Alcanivorax borkumensis*, *Arthrobacter paraffineus*, *Arthrobacter* sp., *Lactobacillus fermentum*, *Nocardia* sp., *Pseudomonas aeruginosa*, *Pseudomonas* sp.  *Rhodococcus erythropolis*, *Rhodotorula glutinous*, *Rhodotorula graminus*, *Serratia marcescens*, *Ustilago maydis* |
|  | Lipopolysaccharides | Yeast: *Candida lipolytica*  Bacteria: *Acinetobacter calcoaceticus*, *Pseudomonas* sp. |
|  | Liposan | Yeast: *Candida lipolytica* |
|  | Mannosylerythritol lipid | Yeast: *Candida antarctica*, *Candida* sp., *Kurtzmanomyces* sp. |
|  | Polyol lipids (liamocins and polyols esters) | Fungus: *Aureobasidium pullulans*  Yeast: *Rhodotorula glutinous*, *Rhodotorula graminus* |
|  | Rhamnolipids | Yeast: *Pseudozyma fusiformata*  Bacteria: *Bacillus subtilis*, *Bacilllus pumilus*, *Bacillus cereu*s, *Pseudomonas aeru*ginosa, *Pseudomonas chlororaphis*, *Pseudomonas fluorescens*, *Pseudomonas putida*, *Pseudomonas* sp., *Renibacterium salmoninarum*, *Serratia rubidea* |
|  | Rubiwettins R1 and RG1 | Bacterium: *Serratia rubidaea* |
|  | Schizonellins A and B | Fungi: *Schizonella melanogramma* |
|  | Sophorolipids | Yeast: *Candida apicola*, *Candida bogoriensis*, *Candida bombicola* (formerly *Torulopsis bombicola*), *Candida lipolytica*, *Torulopsis apicola*, *Torulopsis gropengiesseri*, *Torulopsis magnolia*, *Torulopsis petrophilum*, *Trichosporon asahii*, *Pseudozyma aphidis*, *Pseudozyma fusifornata*, *Pseudozyma rugulosa*, *Wickerhamomyces anomalus*  Bacteria: *Pseudomonas* sp. |
|  | Sucrose and fructose trehalolipids | Bacterium: *Arthrobacter paraffineus* |
|  | Sulfonylipids | Bacterium: *Corynebacterium alkanolyticum*, *Thiobacillus thiooxidans* |
|  | Trehalolipids | Fungus: *Fusarium fujikuroi*  Bacteria: *Acinetobacter* sp., *Arthrobacter* sp., *Arthrobacter paraffineus*, *Brevibacteria* sp., *Corynebacterium* sp., *Mycobacterium* sp., *Mycobacterium tuberculosis*, *Nocardia corynebacterides*, *Nocardia erythropolis*, *Norcardia* sp., *Rhodococcus aurantiacus*, *Rhodococcus erythropolis*, *Rhodococcus ruber*, *Rhodococcus* sps., *Tsukamurella* sp. |
|  | Trehalose dimycolates | Bacterium: *Rhodococcus eryithropolis* |
|  | Ustilipids | Fungi: *Geotrichum candidum*, *Ustilago maydis* |
|  | Monoglucosyloxyoctadecenoic | Fungus: *Aspergillus niger* |
|  | Methoxy phenyl oxime glycosides | Fungus: *Aspergillus flavus* |
|  | Cladosan | Fungus: *Cladosporium resinae* |
|  | Fusaroside | Fungus: *Fusarium sp.* |
| **Lipopeptide and lipoproteins** | Arthrofactin | Bacterium: *Arthrobacter* sp. |
|  | Digalactosyl diglycerides | Bacterium: *Lactobacillus fermentii* |
|  | Gramicidins | Bacterium: *Bacillus brevis* |
|  | Lichenysin | Bacterium: *Bacillus licheniformis* |
|  | Lipoproteins (unspecified) | Bacteria: *Acinetobacter* sp., *Bacillus* sp. |
|  | Lipopeptide (unspecified) | Fungi: *Penicillium chrysogenum*, *Diheterospora chlamydosporia*, *Helminthosporium carbonum*, *Tolypocladium inflatum* (*Trichoderma polysporum*), *Fusarium* SP BS-8  Bacteria: *Achromobacter xylosoxidans*, *Acinetobacter calcoaceticus*, *Acinetobacter* sp., Arthrobacter, *Bacillus licheniformis*, *Bacillus subtilis*, *Micrococcus kristinae*, *Proteus mirabilis*, *Proteus vulgaris*, *Serratia marcescens*, *Sphingomonas paucimobilis*, *Bacillus cereus*, *Bacillus polymyxa*, *Bacillus pumilus*, *Bacillus subtilis*, *Bacillus thuringiensis*, *Pseudomonas fluorescens*, *Pseudomonas putida*, *Streptomyces canus*, *Streptomyces fungicidicus*, *Streptomyces globocacience*, *Streptomyces sioyaensis*, *Thiobacillus thiooxidans* |
|  | Ornithine | Bacteria: *Gluconobacter cerinus*, *Streptomyces sioyaensis*, *Thiobacillus thiooxidans* |
|  | Peptidolipid | Yeast: *Candida petrophilum* |
|  | Polymyxins | Bacterium: *Bacillus polymyxa* |
|  | Serrawettin | Bacterium: *Serratia marcescens* |
|  | Streptofactin | Bacterium: *Streptomyces tendae* |
|  | Subtilisin | Bacterium: *Bacillus subtilis* |
|  | Surfactin | Bacteria*: Bacillus subtilis*, *Bacillus licheniformis*, *Pseudomonas aeruginosa*, *Bacillus licheniformis*, *Bacillus pumilus* |
|  | Viscosin | Bacterium: *Pseudomonas fluorescens* |
| **Neutral lipids** |  | Bacterium: *Nocardia erythropolis* |
| **Ornithinlipids** |  | Bacteria: *Gluconobacter cerinus*, *Pseudomonas rubescens*, *Thiobacillus ferroxidans* |
| **Particulate surfactants (PM)** | Biosur (PM) | Bacterium: *Pseudomonas maltophila* |
|  | Particulate surfactants (unspecified) | Bacteria: *Acinetobacter calcoaceticus*, *Cyanobacteria*, *Pseudomonas marginalis* |
|  | Vesicles and fimbriae | Bacterium: *Acinetobacter calcoaceticus* |
| **Phospholipids** |  | Fungi: *Aspergillus*  Bacteria: *Acinetobacter* sp., *Corynebacterium lepus*, *Thiobacillus thiooxidans* |
| **Polymeric surfactants** | Alasan | Bacterium: *Acinetobacter radioresistens* |
|  | Biodispersan | Bacterium: *Acinetobacter calcoaceticus* |
|  | Carbohydrate-protein-lipid | Fungi: *Cunninghamella echinulate*  Yeast: *Debaryomyces polymorphus*  Bacterium: *Corynebacterium hydrocarboclastus*, *Microbacterium* sp. |
|  | Emulsan | Bacteria: *Acinetobacter calcoaceticus*, *Acinetobacter* sp., *Gordonia*sp. |
|  | Lipomanan | Yeast: *Candida tropicalis* |
|  | Mannan-lipid-protein | Yeast: *Candida tropicalis* |
|  | Protein PA | Bacterium: *Pseudomonas aeruginosa* |
|  | Polymeric Surfactants (unspecified) | Fungus: *Curvularia lunata* IM 2901  Yeast: *Candida lipolytica*, *Candida utilis*  Bacteria: *Acinetobacter calcoaceticus*, *Bacillus stearothermophilus*, *Halomonas eurihalina*, *Mycobacterium thermoautotrophium*, *Sphingomonas paucimobilis* |
|  | Glycoprotein | Fungus: *Aspergillus ustus* MSF3, |
| **Other** | Enamide | Fungus: *Fusarium proliferatum* |
|  | Cerato-platanins | Fungus: *Aspergillus terreus* MUT 271 |
|  | Sap-Pc protein | Fungus: *Penicillium chrysogenum* MUT 5039 |

**References**

1. Wilson, J. B. Methods for fitting dominance/diversity curves. *J. Veg. Sci.* **2**, 35-46; 10.2307/3235896 (1991).

2. Silva, N. R. A. *et al.* Biosurfactant-and-bioemulsifier produced by a promising *Cunninghamella echinulata* isolated from caatinga soil in the northeast of Brazil. *Int. J. Mol. Sci.* **15**, 15377-15395; 10.3390/ijms150915377 (2014).

3. Silva, A. C. S. d., Santos, P. N. d., Silva, T. A. L. e., Andrade, R. F. S. & Campos-Takaki, G. M. Biosurfactant production by fungi as a sustainable alternative. *Arq. Inst. Biol.* **85**; 10.1590/1808-1657000502017 (2018).

4. Cameotra, S. S. & Makkar, R. S. Biosurfactant-enhanced bioremediation of hydrophobic pollutants. *Pure Appl. Chem.* **82**, 97; 10.1351/PAC-CON-09-02-10 (2010).

5. Santos, D. K. F., Rufino, R. D., Luna, J. M., Santos, V. A. & Sarubbo, L. A. Biosurfactants: multifunctional biomolecules of the 21st century. *Int. J. Mol. Sci.* **17**, 401; 10.3390/ijms17030401 (2016).

6. Katemai, W., Maneerat, S., Kawai, F., Kanzaki, H. & Nitoda, T. Purification and characterization of a biosurfactant produced by *Issatchenkia orientalis* SR4. *J. Gen. Appl. Microbiol.* **54**, 79-82; 10.2323/jgam.54.79 (2008).

7. Desai, J. D. & Banat, I. M. Microbial production of surfactants and their commercial potential. *Microbiol. Mol. Biol. Rev.* **61**, 47-64 (1997).

8. Rahman, P. K. S. M. & Gakpe, E. Production, characterisation and applications of biosurfactants-review. *Biotechnol.* **7**, 360-370; 10.3923/biotech.2008.360.370 (2008).

9. Sanches, M. A. *et al.* Production of biosurfactants by *Ascomycetes*. *Int. J. Microbiol.* **2021**, 6669263; 10.1155/2021/6669263 (2021).

10. Ishaq, U. *et al.* Production and characterization of novel self-assembling biosurfactants from *Aspergillus flavus*. *J. Appl. Microbiol.* **119**, 1035-1045; 10.1111/jam.12929 (2015).

11. Elshafie, A. E. *et al.* Sophorolipids production by *Candida bombicola* ATCC 22214 and its potential application in microbial enhanced oil recovery. *Front. Microbiol.* **6**, 1324; 10.3389/fmicb.2015.01324 (2015).

12. Chandran, P. & Das, N. Biosurfactant production and diesel oil degradation by yeast species *Trichosporon asahii* isolated from petroleum hydrocarbon contaminated soil. *Int. J. Eng. Sci. Technol.* **2**, 6942-6953 (2010).

13. Matvyeyeva, O. L. & Aliievа, O. R. Microbial biosurfactants role in oil products biodegradation. *Int. J. Environ. Bioremediat. Biodegrad.* **2**, 69-74; 10.12691/ijebb-2-2-4 (2014).

14. Kitamoto, D. *et al.* Microbial conversion of n-alkanes into glycolipid biosurfactants, mannosylerythritol lipids, by *Pseudozyma* (*Candida antarctica*). *Biotechnol. Lett.* **23**, 1709-1714; 10.1023/A:1012464717259 (2001).

15. Kosaric, N. Biosurfactants and their application for soil bioremediation. *Food Technol. Biotechnol.* **39**, 295-304 (2001).

16. Puntus, I. F., Sakharovsky, V. G., Filonov, A. E. & Boronin, A. M. Surface activity and metabolism of hydrocarbon-degrading microorganisms growing on hexadecane and naphthalene. *Process Biochem.* **40**, 2643-2648; 10.1016/j.procbio.2004.11.006 (2005).

17. Garay, L. A. *et al.* Extracellular fungal polyol lipids: a new class of potential high value lipids. *Biotechnol. Adv.* **36**, 397-414; 10.1016/j.biotechadv.2018.01.003 (2018).

18. Ibrahim, M. L., Ijah, U. J. J., Manga, S. B., Bilbis, L. S. & Umar, S. Production and partial characterization of biosurfactant produced by crude oil degrading bacteria. *Int. Biodeterior. Biodegradation* **81**, 28-34; 10.1016/j.ibiod.2012.11.012 (2013).

19. Das, P., Yang, X.-P. & Ma, L. Z. Analysis of biosurfactants from industrially viable *Pseudomonas* strain isolated from crude oil suggests how rhamnolipids congeners affect emulsification property and antimicrobial activity. *Front. Microbiol.* **5**, 696; 10.3389/fmicb.2014.00696 (2014).

20. Ferhat, S. *et al.* Screening and preliminary characterization of biosurfactants produced by *Ochrobactrum* sp. 1C and *Brevibacterium* sp. 7G isolated from hydrocarbon-contaminated soils. *Int. Biodeterior. Biodegradation* **65**, 1182-1188; 10.1016/j.ibiod.2011.07.013Get (2011).

21. Peng, F., Liu, Z., Wang, L. & Shao, Z. An oil‐degrading bacterium: *Rhodococcus erythropolis* strain 3C‐9 and its biosurfactants. *J. Appl. Microbiol.* **102**, 1603-1611; 10.1111/j.1365-2672.2006.03267.x (2007).

22. Franzetti, A. *et al.* Potential applications of surface active compounds by *Gordonia* sp. strain BS29 in soil remediation technologies. *Chemosphere* **75**, 801-807; 10.1016/j.chemosphere.2008.12.052 (2009).

23. Bustamante, M., Durán, N. & Diez, M. C. Biosurfactants are useful tools for the bioremediation of contaminated soil: a review. *J. Soil Sci. Plant Nutr.* **12**, 667-687; 10.4067/S0718-95162012005000024 (2012).

24. Karlapudi, A. P. *et al.* Role of biosurfactants in bioremediation of oil pollution-a review. *Petroleum* **4**, 241-249; 10.1016/j.petlm.2018.03.007 (2018).

25. Das, N. & Chandran, P. Microbial degradation of petroleum hydrocarbon contaminants: an overview. *Biotechnol. Res. Int.* **2011**; 10.4061/2011/941810 (2011).

26. Ron, E. Z. & Rosenberg, E. Biosurfactants and oil bioremediation. *Curr. Opin. Biotechnol.* **13**, 249-252; 10.1016/S0958-1669(02)00316-6 (2002).

27. Christova, N., Tuleva, B. & Nikolova-Damyanova, B. Enhanced hydrocarbon biodegradation by a newly isolated *Bacillus subtilis* strain. *Z. Naturforsch., C, J. Biosci.* **59**, 205-208; 10.1515/znc-2004-3-414 (2004).

28. Tuleva, B., Christova, N., Jordanov, B., Nikolova-Damyanova, B. & Petrov, P. Naphthalene degradation and biosurfactant activity by *Bacillus cereus* 28BN. *Z. Naturforsch., C, J. Biosci.* **60**, 577-582; 10.1515/znc-2005-7-811 (2005).

29. Xia, W. *et al.* Biosurfactant produced by novel *Pseudomonas* sp. WJ6 with biodegradation of n-alkanes and polycyclic aromatic hydrocarbons. *J. Hazard. Mater.* **276**, 489-498; 10.1016/j.jhazmat.2014.05.062 (2014).

30. Banat, I. M., Rahman, K. S. M. & Thahira-Rahman, J. Bioremediation of hydrocarbon pollution using biosurfactant producing oil degrading bacteria. *WIT Trans. Ecol. Environ.* **59**; 10.2495/OIL020201 (2002).

31. Bodour, A. A. *et al.* Structure and characterization of flavolipids, a novel class of biosurfactants produced by *Flavobacterium* sp. strain MTN11. *Appl. Environ. Microbiol.* **70**, 114-120; 10.1128/AEM.70.1.114-120.2004 (2004).

32. Al-Hawash, A. B., Zhang, X. & Ma, F. Removal and biodegradation of different petroleum hydrocarbons using the filamentous fungus *Aspergillus* sp. RFC-1. *MicrobiologyOpen* **8**, e00619; 10.1002/mbo3.619 (2019).

33. Pinedo-Rivilla, C., Aleu, J. & Collado, I. G. Pollutants biodegradation by fungi. *Curr. Org. Chem.* **13**, 1194-1214; 10.2174/138527209788921774 (2009).

34. Gargouri, B., Mhiri, N., Karray, F., Aloui, F. & Sayadi, S. Isolation and characterization of hydrocarbon-degrading yeast strains from petroleum contaminated industrial wastewater. *BioMed Res. Int.* **2015**; 10.1155/2015/929424 (2015).

35. Ilori, M. O., Adebusoye, S. A. & Ojo, A. C. Isolation and characterization of hydrocarbon-degrading and biosurfactant-producing yeast strains obtained from a polluted lagoon water. *World J. Microbiol. Biotechnol.* **24**, 2539-2545; 10.1007/s11274-008-9778-3 (2008).

36. Bento, F. M., Camargo, F. A. d. O., Okeke, B. C. & Frankenberger Jr., W. T. Diversity of biosurfactant producing microorganisms isolated from soils contaminated with diesel oil. *Microbiol. Res.* **160**, 249-255; 10.1016/j.micres.2004.08.005 (2005).

37. Bicca, F. C., Fleck, L. C. & Ayub, M. A. Z. Production of biosurfactant by hydrocarbon degrading *Rhodococcus ruber* and *Rhodococcus erythropolis*. *Rev. Microbiol.* **30**, 231-236; 10.1590/S0001-37141999000300008 (1999).

38. Ayed, H. B. *et al.* Enhancement of solubilization and biodegradation of diesel oil by biosurfactant from *Bacillus amyloliquefaciens* An6. *Int. Biodeterior. Biodegradation* **99**, 8-14; 10.1016/j.ibiod.2014.12.009 (2015).

**Supplementary Video Legends**

Video 1: BS activity 1.mp4

Legend: Oil-spreading assay showing presence of biosurfactant(s). One example isolate is shown to demonstrate the presence of biosurfactant(s) confirmed by oil displacement activity where there is a clearing zone like a halo forming.

Video 2: BS activity 2.mp4

Legend: Oil-spreading assay showing presence of biosurfactant(s). One example isolate is shown to demonstrate the presence of biosurfactant(s) confirmed by oil displacement activity where there is a clearing zone like a halo forming.

Video 3: Negative control 1.mp4

Legend: Rep 1 of the negative control showing no detection of biosurfactant(s).

Video 4: Negative control 2.mp4

Legend: Rep 2 of the negative control showing no detection of biosurfactant(s).
